# Integrated analysis of small RNAs, transcriptome and degradome sequencing reveal the drought stress network in *Agropyron mongolicum* Keng

**DOI:** 10.3389/fpls.2022.976684

**Published:** 2022-08-17

**Authors:** Bobo Fan, Fengcheng Sun, Zhuo Yu, Xuefeng Zhang, Xiaoxia Yu, Jing Wu, Xiuxiu Yan, Yan Zhao, Lizhen Nie, Yongyu Fang, Yanhong Ma

**Affiliations:** ^1^Agricultural College, Inner Mongolia Agricultural University, Hohhot, China; ^2^Inner Mongolia Academy of Agricultural & Animal Husbandry Sciences, Hohhot, China; ^3^College of Grassland, Resources and Environment, Inner Mongolia Agricultural University, Hohhot, China

**Keywords:** *Agropyron mongolicum* Keng, drought resistance, microRNAs, transcriptome, degradome, integration analysis, co-expression network

## Abstract

*Agropyron mongolicum* (*A. mongolicum*) is an excellent gramineous forage with extreme drought tolerance, which lives in arid and semiarid desert areas. However, the mechanism that underlies the response of microRNAs (miRNAs) and their targets in *A. mongolicum* to drought stress is not well understood. In this study, we analyzed the transcriptome, small RNAome (specifically the miRNAome) and degradome to generate a comprehensive resource that focused on identifying key regulatory miRNA-target circuits under drought stress. The most extended transcript in each collection is known as the UniGene, and a total of 41,792 UniGenes and 1,104 miRNAs were identified, and 99 differentially expressed miRNAs negatively regulated 1,474 differentially expressed target genes. Among them, eight miRNAs were unique to *A. mongolicum*, and there were 36 target genes. A weighted gene co-expression network analysis identified five hub genes. The miRNAs of five hub genes were screened with an integration analysis of the degradome and sRNAs, such as osa-miR444a-3p.2*-MADS47*, bdi-miR408-5p_1ss19TA-*CCX1*, tae-miR9774_L-2R-1_1ss11GT*-carC*, ata-miR169a-3p*-PAO2*, and bdi-miR528-p3_2ss15TG20CA*-HOX24*. The functional annotations revealed that they were involved in mediating the brassinosteroid signal pathway, transporting and exchanging sodium and potassium ions and regulating the oxidation–reduction process, hydrolase activity, plant response to water deprivation, abscisic acid (ABA) and the ABA-activated signaling pathway to regulate drought stress. Five hub genes were discovered, which could play central roles in the regulation of drought-responsive genes. These results show that the combined analysis of miRNA, the transcriptome and degradation group provides a useful platform to investigate the molecular mechanism of drought resistance in *A. mongolicum* and could provide new insights into the genetic engineering of Poaceae crops in the future.

## Introduction

Global warming increases the environmental stress on crops, making it difficult to fully utilize their genetic potential (Boyer, 1982). The total yield of principal crops decreases by approximately 70% each year owing to ecological pressures (Shubha and Tyagi, 2010; Zurbriggen et al., 2010). Among all the ecological pressures, the impact and restriction of drought on agriculture are particularly prominent (Esfahanian et al., 2017). However, plants that grow in some barren and arid environments have evolved highly effective self-regulatory systems to manage drought. Plants of the *Agropyron* genus are significant pasture resources that have adapted to drought, low temperature and salinity and play essential roles in ecological restoration (Che and Li, 2007; Han et al., 2017). *Agropyron mongolicum* (Poaceae, 2*n* = 2*x* = 14), a representative member of *Agropyron*, is an excellent perennial grazing grass in the arid steppe, which has soft stems and leaves, a highly developed root system, early greening, strong tillering ability, good palatability, adaptability, and is known for its strong drought resistance (Zhao et al., 2010b; Zhang et al., 2019). It can be used for pasture and ecological restoration in cold and arid regions and provides superior gene resources to breed drought resistance and improve forage and wheat (*Triticum aestivum*) crops (Du et al., 2017a). However, the molecular drought resistance mechanism of *A. mongolicum* is still in its infancy and merits more attention and urgent research work.

Drought can cause physiological and morphological changes in plants, which negatively affect their growth and productivity (Chandra et al., 2021). MicroRNAs (miRNAs) play crucial roles in plant-environment interactions (Niu et al., 2016; Song et al., 2019). miRNAs range from 18 to 25 nucleotides (nt) and are a class of endogenous small non-coding RNAs (Carrington and Ambros, 2003; Song et al., 2019). miRBase (miRbase V22.1), a professional research database of miRNA, contains 38,589 mature miRNAs of 271 species, which include 9,168 mature miRNAs of 74 dicotyledonous and monocotyledonous plants. On the basis of sequence complementarity, miRNAs directly target mRNAs to cleave or translationally repress them, thus, completing the regulation of plant function (Frank et al., 2000; Flynt and Lai, 2008). Currently, miRNAs involved in abiotic stress (drought, salinity and temperature) responses have been reported in *Arabidopsis thaliana*, tobacco (*Nicotiana tabacum*), rice (*Oryza sativa*) and other plants. They include miR156, miR159, miR169, miR395 (Ali et al., 2017), miR444 (Jiao et al., 2020; Kannan et al., 2022), miR408 (Hajyzadeh et al., 2015; Taier et al., 2021), and miR528 (Chen et al., 2021; Wang et al., 2021a). miRNAs regulate transcription factors, plant hormones, antioxidant systems and other functional target genes, such as *MYB33*, *OsNAC2*, *ARF22*, *DREB*, *NtCAT1*, *ARF*, *TIR1*, and *OsMADS27* (Reyes and Nam-Hai, 2007; David and Franck, 2011; Hajyzadeh et al., 2015; Yin et al., 2015; Jiang et al., 2018; Qiu et al., 2018; Shi et al., 2018; Kannan et al., 2022) to respond to abiotic stress.

miRNA-led stress regulatory networks are considered novel tools for the development of abiotic stress tolerance in crops (Ali et al., 2017). The integrated miRNAs-target genes interaction network can intuitively reflect the relationship between genes at the global level. The hub genes are the core regulatory genes in the network and play a crucial role in the stability of network. The integrated analysis of miRNA and target mRNAs has been widely used in human medicine, animal growth and development, gene expression regulatory mechanisms and other fields, but research reports on its use in plants are limited. The general workflow for constructing microRNA-mediated gene regulatory network was introduced (Meng et al., 2011). The regulatory network of auxin, miR390, *TAS3* and *ARFs* on the root growth of *Arabidopsis thaliana* (*A. thaliana*) was analyzed (Elena et al., 2010). Floral transcriptomes in woodland strawberries (*Fragaria vesca*) uncovered developing receptacles and anther gene networks and identified the hub genes *FveLOM* and *FveWUS1* (Hollender et al., 2014). A cadmium phytoremediation miRNA-target mRNA network was constructed in hyperaccumulating *Sedum alfredia*, and the hub genes *AAP3* and *ARF4* may play a key regulatory role (Han et al., 2016). Two cold-resistant modules of peanut (*Arachis hypogeae*) were found by a weighted gene co-expression network analysis (WGCNA). They obtained the hub genes involved in soluble sugar, polyamine and the G-lignin biosynthetic pathway at low temperatures (Wang et al., 2021c). A total of 13 hub genes were identified in cotton (*Gossypium hirsutum*) in which the expression of *Gh_A06G1257* was significantly the highest in different tissues, and it was identified as regulating drought stress by a gene silencing technique (Gereziher et al., 2021). The hub gene and regulatory network of cotton were discovered under salt and drought stress (Bano et al., 2022). The research model of gene interaction networks can explore hub genes and the complex regulatory network of drought and salt stress. In this study, the drought-related modules were studied based on the transcriptome data to obtain a comprehensive drought gene regulatory network, which is conducive to the in-depth understanding and analysis of the function of hub genes in complex networks.

The level of tolerance of plants to drought conditions is coordinated by the action of different drought-responsive genes about other stress components, such as high temperature and salt stress, which stimulate signal transduction pathways and are complex and mutagenic (Oladosu et al., 2019; Gereziher et al., 2021). Exploring the molecular mechanism of drought resistance in *A. mongolicum* requires a comprehensive analytical method, and the current high-throughput sequencing technology provides an effective platform (Mutz et al., 2013; Han et al., 2016). The integrated analysis of transcriptome, sRNAs and degradome offers the feasibility to select and identify the drought stress gene regulatory network and hub genes of *A. mongolicum*.

The regulatory mechanism of drought resistance and hub genes in *A. mongolicum* is still in its early stages. The regulation of drought resistance is complicated, which restricts the development and utilization of gene resources in response to drought stress. By integrating the transcriptome, sRNAs, and degradome analysis, this study aimed to identify the hub genes of drought resistance and establish a co-expression regulatory network of hub genes in *A. mongolicum*. The findings are meaningful to identify drought resistance hub genes and understand the molecular mechanism of drought resistance in *A. mongolicum*, which should promote genetic engineering research on drought resistance in Poaceae crops.

## Materials and methods

### Plant materials and drought stress treatment

Mature seeds of *A. mongolicum* were collected from the Inner Mongolia Agricultural University’s (Hohhot, China) forage test station in the Inner Mongolia Autonomous Region. The lemma was removed from the intact seeds, which were disinfected with a (1,1.3) sodium hypochlorite solution (v/v) for 15 min and then rinsed five times with sterilized distilled water. The seeds were planted in a germination box and cultured in a controlled climate growth chamber at 24°C with 16 h of daylight with an illumination intensity of 30,000 Lx (BIC-300; Boxun, Shanghai, China). Sterilized distilled water was sprayed quantitatively during the seed germination. When the seedlings grew to be 5–8 cm high, they were transplanted into a germination box with 20% Hoagland’s nutrient solution and cultured until the three-leaf one heart phase. The seedlings were then exposed to a drought treatment with 25% polyethylene glycol (PEG)-6,000. Their leaves were sampled at 0 h (CK), 12 h (D_12 h), 24 h (D_24 h), 48 h (D_48 h), 3 days (D_3 days), 5 days (D_5 days), and 7 days (D_7 days), and rewatered at 24 h (R_24 h). The sample weight of each biological replicate was 1 g of fresh leaves. The samples were frozen in liquid nitrogen and stored at −80°C.

### Total RNA extraction

The total RNA was extracted from the samples using the TRIzol reagent (Invitrogen, Carlsbad, CA, United States). The quantity and purity of RNA were evaluated using a NanoDrop ND-1000 (Thermo Fisher Scientific, Waltham, MA, United States), and the integrity and concentration of RNA were assessed using an Agilent Bioanalyzer 2,100 (Agilent Technology, Santa Clara, CA, United States; RIN number > 7.0, OD_260/280_ > 1.8). The total RNA collected from each treatment and control group was utilized to create the library and transcriptome, sRNA and degradome sequencing. The samples were sequenced *de novo* by Hangzhou LC Biology Co., Ltd. (Hangzhou, China).

### Transcriptome sequencing and *de novo* assembly analysis

Certified total RNA was purified twice using poly-T oligo linked magnetic beads to obtain poly (A) RNA. Following purification, the mRNA was fragmented into minute fragments under increased temperature using divalent cations. The cleaved RNA fragments were then reverse-transcribed to form the final cDNA library, which was consistent with the manufacturer’s instructions for the RNA-Seq sample preparation kit (Illumina, San Diego, CA, United States), with an average insert size of 300 bp (±50 bp) for the paired-end libraries. The paired-end samples were sequenced on an Illumina HiSeq 6,000 at LC Sciences (Houston, TX, United States) according to the manufacturer’s instructions.

Cutadapt (Martin, 2011) and in-house Perl scripts were used to delete the reads with adapter contamination, low-quality bases, and uncertain bases. The sequence quality was then confirmed using FastQC,^1^ which included the Q20, Q30, N50 and GC content of the clean data. All the downstream analyses relied on high quality clean data. Trinity 2.4.0^2^ was used to accomplish *de novo* transcriptome assembly (Grabherr et al., 2011). Trinity groups transcripts into clusters, and the most extended transcript in each collection is known as the UniGene.

All the assembled UniGenes were aligned against the non-redundant (Nr) protein database,^3^ Gene Ontology (GO),^4^ SwissProt,^5^ Kyoto Encyclopedia of Genes and Genomes (KEGG)^6^ and eggNOG^7^ databases using DIAMOND (Buchfink et al., 2014) with a threshold of *E*-value < 0.00001.

### Differentially expressed unigene analysis

Salmon (Patro et al., 2017) was used to identify the levels of expression for UniGenes by calculating the transcript per million (TPM; Mortazavi et al., 2008). The differentially expressed unigenes were selected with log_2_ (fold change) ≥1 or log_2_ (fold change) ≤ −1 and with the statistical significance of *p* ≤ 0.05 by the R package edgeR (Smyth, 2010). GO and KEGG enrichment analyses were then performed on the differentially expressed genes (DEGs) using in-house Perl scripts.

### sRNA sequencing and miRNA basic data analysis

A TruSeq Small RNA Sample Prep Kit (Illumina) was used to create the miRNA library. The experiment was conducted according to the manufacturer’s instructions. An Illumina HiSeq 2,500 platform was used to sequence the cDNA library, and single-end (SE50) sequencing was utilized (50 bp). To obtain clean reads, raw reads were submitted to an in-house tool designated ACGT101-miR (LC Sciences), which removed adaptor dimers, repetitions, junk sequences and typical RNA families (rRNA, tRNA, snRNA, and snoRNA). The subsequent use of a BLAST search mapped unique sequences that were 18–25 nt long to miRBase 22.0 to identify the miRNAs. The miRNAs matched to the database are known, while those that do not map to it are unique miRNAs in *A. mongolicum*.

To better understand the overall evolutionary relationship and conservation of miRNA, the family classification of miRNA, the frequency of miRNA in other species, the number of miRNA precursors in different species and the base preference of miRNA were statistically analyzed.

### Analysis of the differentially expressed miRNAs

Based on the experimental design, the differential expression of miRNAs was evaluated using a *t*-test. The significance threshold was set at 0.01 and 0.05 in each test. Differentially expressed miRNAs were assigned a *value of p* ≤ 0.05 and a|log_2_ (fold change)| ≥ 1, and a cluster diagram of the miRNA expression (norm value) was constructed. The up- and downregulated miRNAs were simultaneously enumerated from differentially expressed miRNAs.

### Degradome sequencing, target identification and analysis

The total RNA (20 μg) was purified using poly-T oligo-attached magnetic beads (Thermo Fisher Scientific), and the mRNA was collected using poly (A). The captured mRNA was coupled using 5′ adaptor primers (Genewiz, Plainfield, NJ, United States). The attached product was transferred into a new centrifuge tube and supplemented with KAPA Pure Beads (kk8000; Roche Diagnostics Application, Indianapolis, IN, United States). The reverse transcription was amplified with reverse transcriptase (m0368; New England Biolabs, Ipswich, MA, United States). The degradation group sequencing library was built with an NEBNext Ultra II RNA Library Prep Kit (e7770; New England Biolabs). Single-ended sequencing was performed using an Illumina HiSeq 2,500 (Hangzhou LC Biology Co., Ltd.) as previously described (Ma et al., 2010).

The target gene was predicted using the Cleaveland 4.0 algorithm (Addo-Quaye et al., 2009), and oligomap was used to accurately match the mRNAs from different species to the *A. mongolicum* degradation group sequence (Berninger et al., 2008). The redundant sequences were removed from the effective data using norm reads per million (NRPM). The Needle program in EMBoss was used to collect all the sequences that matched the target genes in the miRNA library, and the target genes were scored according to the plant miRNA/target pairing standard (Allen et al., 2005). In addition, the degradome reads were mapped to the *A. mongolicum* transcriptome data as previously described (Xu et al., 2013; Yang et al., 2013). The number of unique genes and the mapping ratio between ideal mRNA and degradation group fragments were counted to assess the degradation group sequencing.

### Integrated analysis of transcriptome, miRNA and degradome sequencing

All the miRNAs and their target genes were obtained based on an integrated analysis of the transcriptome, miRNA, and degradome group data. The connection pairs between differential miRNAs and their differential target genes were created using a threshold of *p* ≤ 0.05. The miRNAs and their target gene pairings with negative regulatory relationships were chosen. The stats package in the *R package* (version 3.6.1) was used to calculate the cluster data, and the heatmap software (version 1.0.12) was used to create a cluster heat map for the DEGs in negative regulatory mode. The transcription function was annotated.

### Construction of the gene co-expression network and screening of the hub gene

A co-expression network analysis was performed based on the transcriptome data using the WGCNA R package (version 1.69). The unigenes of missing values were removed, and the expression data of mRNA was regenerated after filtering. The sample clustering tree was then constructed; the outlier samples were removed, and the appropriate scale-free key parameter *β* was selected. To ensure a scale-free network, power = 4 was chosen to build a systematic clustering tree between the genes. All the other settings were left at the default levels, including the module size of gene modules, the number of modules, computing the feature vector value between modules, and performing module clustering analysis. The webpage of the Horvath lab^8^ contains a thorough description of WGCNA that explains the analytical stages (Langfelder and Horvath, 2008).

The module constructed by WGCNA and drought-related physiological indicators were used for correlation analysis to select specific modules related to drought resistance. The connectivity top 20 in the module were defaulted to be hub genes. The candidate hub genes related to drought resistance were screened from the high connective and specificity genes by GO enrichment analysis. The targeted relationships between the hub genes and miRNAs were confirmed by degradome sequencing data. The drought resistance candidate hub genes of the *A. mongolicum* regulatory network were mapped using Cytoscape (version 3.9.1).

### RT-qPCR analysis of miRNA and potential drought resistant candidate hub genes

Quantitative reverse transcription PCR (RT-qPCR) was performed on selected miRNA and candidate hub genes related to drought resistance. Primer Premier 6.0 software was used to design the primers, and the primers were synthesized by Sangon Biotech Co., Ltd. (Shanghai, China; Table 1). An miRcute Plus miRNA qPCR Detection Kit (FP411) and an miRcute Plus miRNA First-Strand cDNA Kit (KR211) from TianGen (Beijing, China) were used for reverse transcription and RT-qPCR of the miRNA. A FastQuant RT Kit (KR106; TianGen) and MonAmp SYBR Green qPCR Mix (MQ10201S; Monad Biotech Co., Ltd.) were used for reverse transcription and RT-qPCR of the candidate hub genes. The relative levels of expression were calculated by the 2^ ^–ΔΔct^ method using U6 as internal standards (Livak and Schmittgen, 2001).

**Table 1:** A list of primers used for validation in RT-qPCR. RT-qPCR, quantitative reverse transcription PCR.

Primer name	Primer sequence
F-osa-miR444a-3p.2	GTGCTGTTGCTGCCTCATGCTT
F-bdi-miR528-p3_2ss15TG20CA	GGTCTTCCATTCCTGCGGCTAA
F-bdi-miR408-5p_1ss19TA	TTCCCTGCAAGCACTTCACG
F-ata-miR169a-3p	TGGGCATGTCAGCGTCGCTAC
F-tae-miR9774_L-2R-1_1ss11GT	GGGGGCGGACTTATTGTGTATATCTGA
F-MADS47(TRINITY_DN16091_c0_g9)	CAGGCTCGGATTACCACTCTTCAAC
R-MADS47(TRINITY_DN16091_c0_g9)	TATTCAATGCAATGCGCGGTTCAAC
F-HOX24(TRINITY_DN19559_c1_g1)	CCGCCCACGACTTCCATTTCTAC
R-HOX24(TRINITY_DN19559_c1_g1)	CTGCTGCTGGTGCTGCTTGG
F-CCX1(TRINITY_DN29080_c0_g1)	ATCTAGTGACGGAGCAGCAGTACC
R-CCX1(TRINITY_DN29080_c0_g1)	GCAAAGCACCAAATCGTGTCTGTC
F-PAO2(TRINITY_DN28296_c0_g1)	ACAAGTGGCGACAACTCTGTTCTG
R-PAO2(TRINITY_DN28296_c0_g1)	AACTTTAGCAGCCGTCTCCATTGG
F-carC(TRINITY_DN28552_c0_g1)	GTCCAGGGCAATTTAGGTAACATTTCG
R-carC(TRINITY_DN28552_c0_g1)	TCCGACGAGGCGATGATGATATTAAC

## Results

### Transcriptome sequencing in *Agropyron mongolicum* under drought treatments

To profile the expression of genes in *A. mongolicum* in response to drought stress, eight libraries were constructed from leaf samples (CK: control, D_12 h, D_24 h, D_48 h, D_3 d, D_5 d, D_7 d: plants under drought stress, R_24 h: plants rewatering after drought stress). More than 145.27 Gb raw reads were generated, and 139.46 Gb clean sRNA reads were obtained. The average Q20 of the sample was 97.445%. Each raw read and clean read of each sample were tallied (Supplementary Table 1A). After quality control, the transcripts were assembled into 41,792 unigenes, and the unigene lengths ranged from 201 bp to 16,861 bp (Table 2; Supplementary Figure 1).

**Table 2:** Summary of transcriptome sequencing for *Agropyron mongolicum.*

Index	*A. mongolicum*
Raw reads (Gb)	145.27
Clean reads (Gb)	139.46
Number of unigenes	41,792
GC (%)	50.34
Average Q20 (%)	97.445
Minimum length (bp)	201
Median length (bp)	704
Maximum length (bp)	16,861

### Functional annotation and enrichment analysis of the unigenes

**Figure 1:**
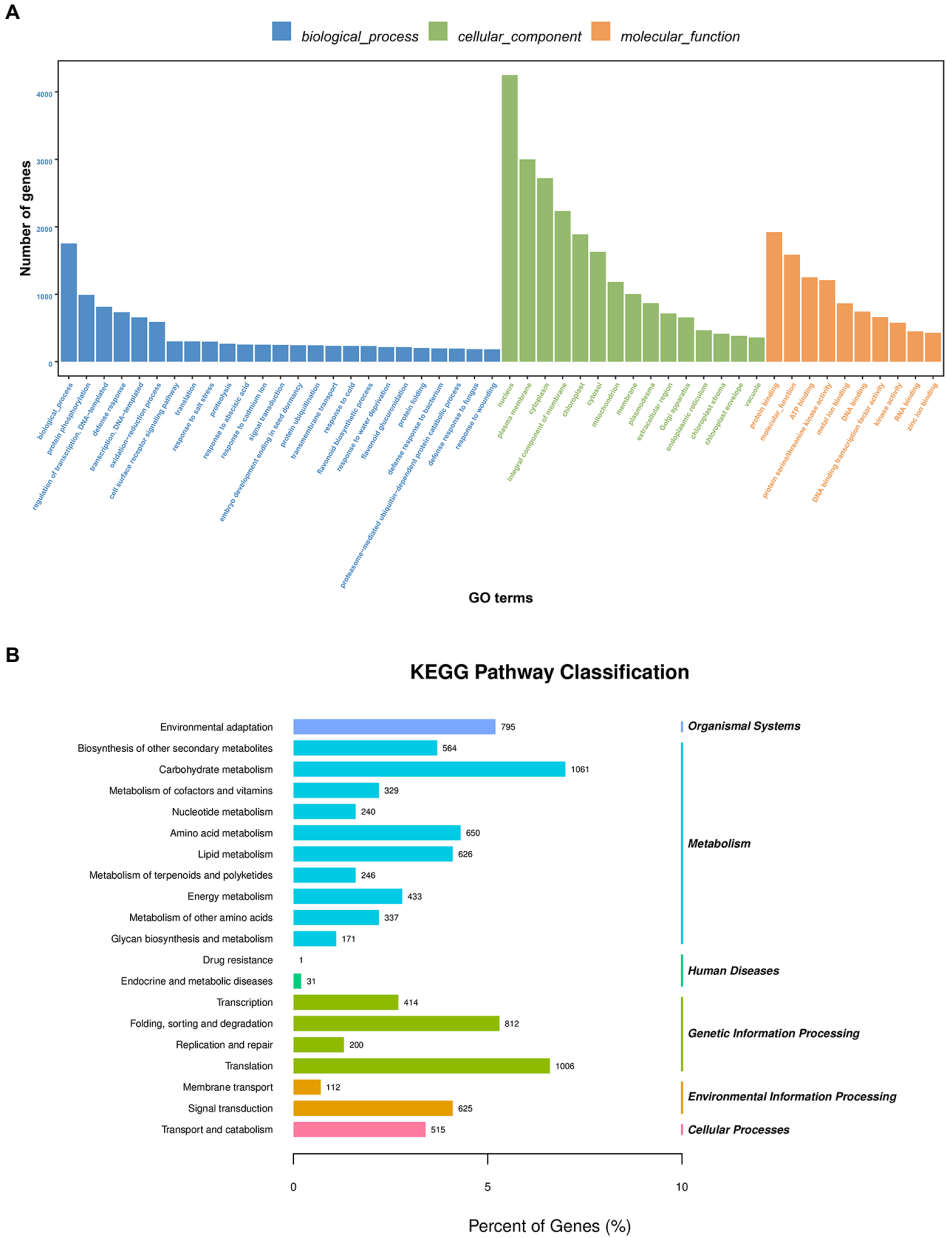
GO classification **(A)** and KEGG pathway enrichment **(B)** of UniGenes. GO, Gene Ontology; KEGG, Kyoto Encyclopedia of Genes and Genomes.

GO functional annotation and enrichment were performed on the transcriptome sequenced UniGenes. A total of 18,259 UniGenes were found to be enriched based on the GO analysis, which accounts for 43.69% of all the UniGenes (Supplementary Table 1B). These terms were primarily involved in biological processes, cell component and molecular function. The GO terms included response to salt stress, response to water deprivation, and response to wounding (Figure 1A) according to the study of GO enrichment in *A. mongolicum*. The enrichment of transcripts was analyzed using KEGG. The transcripts were primarily enriched in five significant pathways, including organic system, metabolism, genetic information processing, environmental information processing and cellular processes. The main pathways involved carbohydrate metabolism, amino acid metabolism and lipid metabolism. In addition, they also participated in translation, folding, sorting and degradation, transcription, replication and repair, signal transduction, membrane transport, transport and catabolism (Figure 1B).

### Analysis of the differentially expressed genes

**Figure 2:**
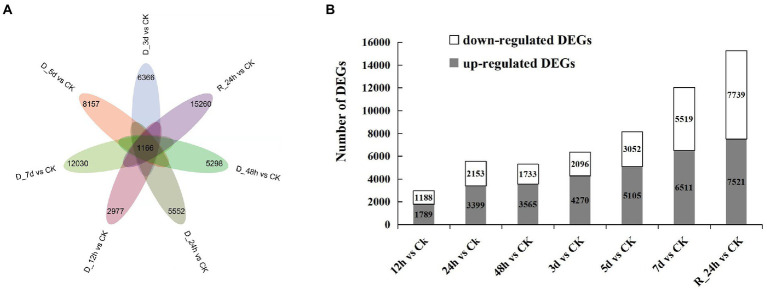
Drought responsive genes in *Agropyron mongolicum*. **(A)** Venn diagrams of differentially expressed genes in the treatment and control groups. **(B)** The number of differentially expressed genes under drought stress compared with the control.

In contrast to the control group, the number of DEGs was screened in each comparison group (Figures 2A,B). A total of 1,166 DEGs were obtained in the seven comparison groups (D_12 h vs. CK, D_24 h vs. CK, D_48 h vs. CK, D_3 d vs. CK, D_5 d vs. CK, D_7 d vs. CK, and R_24 h vs. CK). The number of DEGs generally increased as the drought lasted longer, while the number of DEGs reduced in a comparison group (48 h vs. CK). After 12 h of drought treatment, there were 1,789 upregulated genes and 1,183 downregulated genes compared with the control. There were 3,399 upregulated genes and 2,153 downregulated genes compared with the CK after 24 h of drought treatment. The number of upregulated DEGs was 3,565, 4,270, 5,105, 6,511, and 7,521 compared with the CK at 48 h, 3 d, 5 d, 7 d, and R_24 h under drought treatment, respectively, whereas the number of downregulated DEGs was 1,733, 2,096, 3,052, 5,519, and 7,739, respectively. The information on all the DEGs in seven comparisons is shown in Supplementary Table 2.

### Sequencing and identification of the miRNAs

The number of known mature miRNA families of *A. mongolicum* was 71 (Supplementary Figure 2A). In contrast, the pre-miRNA sequences of *A. mongolicum* were highly similar to the known pre-miRNAs in 46 plant species (Supplementary Figure 2B). All the miRNAs were categorized into four groups (gp1, gp2, gp3, and gp4) based on their abundance in the miRNA database and sequencing reads (Supplementary Table 3). miRNA sequences that matched the pre-miRNA sequences in the miRNA database were classified as known miRNAs, whereas those that did not were classified as novel miRNA candidates of *A. mongolicum*. A total of 895 mature miRNAs had been obtained, and the length of known miRNA sequences ranged from 18 to 25 nt, with those that were 21 nt accounting for 49.50%. There were 209 novel miRNAs, and the sizes ranged from 19 to 24 nt, accounting for 77.51% at 21 nt (Supplementary Tables 4, 5A; Supplementary Figures 2C,D). The minimal folding free energy index (MFEI) offers a standard for comparing the MFE of pre-miRNA of different lengths of *A. mongolicum*. The minimal folding energy (MFE) is related to the sequence length of pre-miRNA (Zhang et al., 2006). The MFEI of the novel miRNA in *A. mongolicum* ranged from 0.90 to 2.30 kcal/mol (Supplementary Table 6). It was indicative of the production of a stable hairpin structure.

### Differentially expressed miRNAs under drought stress

**Figure 3:**
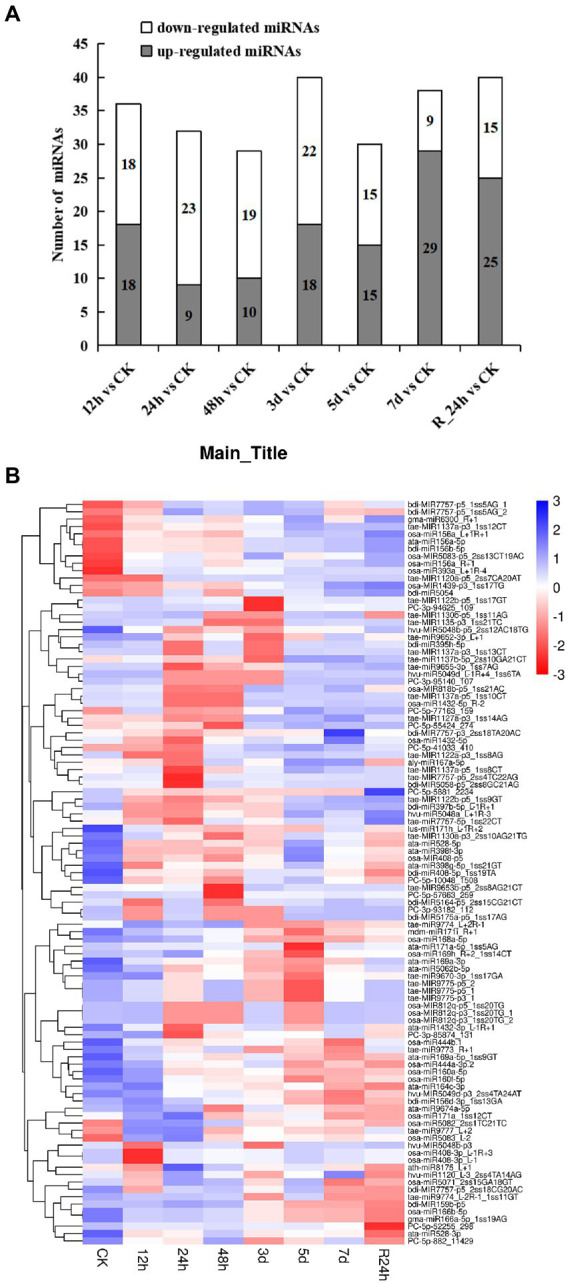
Differentially expressed miRNAs under drought stress. **(A)** The number of differentially expressed miRNAs compared with the control. **(B)** Hierarchical clustering of differentially expressed miRNAs in eight different durations of drought treatment [CK (0 h), 12 h, 24 h, 12 h, 48 h, 3 days, 5 days, 7 days, and R24 h]. Red indicates a lower level of expression of the miRNAs, and blue indicates higher levels of expression of miRNAs. The names of the samples are shown at the bottom, and the name of the differentially expressed gene is shown on the right. The norm expression of miRNA was standardized using a Z-score. The absolute signal intensity ranges from −3 to +3, and the corresponding color changes from red to blue.

A total of 101 differentially expressed miRNAs (*p* ≤ 0.05) were found among the 24 libraries. The distribution of differential expression miRNAs between the control and treatments was examined (Figure 3A). Compared with the control, the number of upregulated genes (29 miRNAs) was the largest at 7 days under drought stress, and the number of downregulated genes (23 miRNAs) was the largest at 24 h under drought stress. To analyze 101 differentially expressed miRNAs of *A. mongolicum* under drought stress, including 12 novel and 89 known miRNAs (Supplementary Table 7), a clustering diagram was generated based on the normal values of miRNA expression (Figure 3B). The expression of miRNAs in the same family was similar under drought stress treatment. The miR156 family, such as osa-miR156_L+R+1, ata-miR156a-5p, and bdi-miR156b-5p, were significantly upregulated under drought treatment. The expression of miR1137 family was upregulated at 5 days, 7 days, and R_24 h. The expression of miR166 family, including osa-miR166b-5p and gma-miR166a-5p_1ss19AG, was downregulated. Among the 12 differentially expressed novel miRNAs, PC-5p-5881_2,234 was downregulated under drought treatment and upregulated at R_24 h. In general, the level of expression of the four novel miRNAs, which included PC-5p-10048_1508, PC-5p-52255_298, PC-5p-882_11429, and PC-3p-85874_131, were downregulated to different degrees at 12 h to 7 days under drought treatment, and their levels of expression were downregulated at 24 h of rehydration. The levels of expression of four novel miRNAs (PC-3p-93182_112, PC-3p-94625_109, PC-5p-57663_259, and PC-3p-95140_107) were upregulated at 5 and 7 days under drought stress and rehydration at 24 h. Three novel miRNAs (PC-5p-55424_274, PC-5p-41033_410, and PC-5p-77163_159) were downregulated at 24 h under drought stress and upregulated from 3 days under drought stress to 24 h of rehydration (Figure 3B). These data suggest that the expression of miRNAs changes with the prolongation of drought stress and do not always remain up- or downregulated.

### Degradome sequencing analysis

After removing the unqualified sequences, 28,885, 26,523, 17,691, 31,196, 28,063, 40,389, 35,357, and 38,874 unique reads were obtained from the CK and each drought treatment group (Supplementary Table 5B), respectively. The ratios of acquired unique mapped reads were 46.83, 53.01, 52.98, 53.63, 53.30, 56.17, 57.03, and 54.35%, respectively (Supplementary Table 5B). The ratios of covered transcripts in the five treatment groups were 80.80, 80.01, 82.18, 81.81, and 81.40%, respectively. The ratios of covered transcripts in the control and drought treatments were 76.52, 77.87, and 71.96% at CK, 12 h, and 24 h of drought stress, respectively. The target genes were separated into five categories (Category 0, 1, 2, 3, and 4; Xu et al., 2013; Yang et al., 2013). A total of 90, 71, 2,591, 2,758, and 3,972 target genes of miRNAs in *A. mongolicum* were in each category (Supplementary Table 5C). A total of 676 miRNAs that regulate 46,456 target genes were identified by degradome sequencing (Supplementary Table 8), and 137 novel miRNAs regulated 1,394 target genes (Supplementary Table 9).

### Integration analysis of miRNAs and their target genes under drought stress

**Figure 4:**
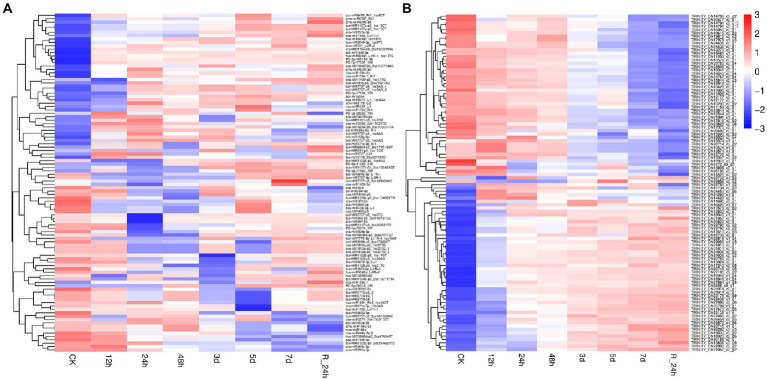
A combined view of the levels of expression between differentially expressed miRNAs **(A)** and their target genes **(B)** in *Agropyron mongolicum* in eight different drought treatment durations. The original expression values of miRNAs and their target genes were normalized by Z-score normalization.

To investigate the trends of miRNA and mRNA changes in *A. mongolicum* under different stages of drought stress, the differentially expressed miRNAs and DEGs from miRNA were integrated and analyzed based on transcriptome and degradome sequencing data. The miRNAs and their target genes with negative regulatory mode were screened in the control group compared with the seven drought-treated groups based on the general model (cleavage) of the miRNA regulation of target genes in plants (Llave et al., 2002). A total of 91 known miRNAs that belonged to 59 miRNA families were found to negatively regulate 1,438 target genes (Figures 4A,B; Supplementary Table 10), indicating that the miRNAs and target genes are not all regulated one-to-one but are regulated in a network-like radial pattern. One of the target genes (norm value of the highest) was chosen to participate in the heatmap when one miRNA regulated many target genes (Figures 4A,B). Eight differentially expressed novel miRNAs of *A. mongolicum* regulated 36 target genes. Seven upregulated the expression of miRNAs, such as PC-3p-105150_88, PC-3p-68960_194, PC-3p-77094_159, PC-5p-41033_410, PC-5p-77163_159, PC-5p-78312_155, and PC-5p-87000_128, and PC-3p-85874_131 was a downregulated miRNA (Figure 4A; Supplementary Table 11). A total of 36 target genes under drought stress were annotated as uncharacterized oxidoreductase, protein MAK16 homolog, magnesium-chelatase subunit ChlH chloroplastic, NADP-thioredoxin reductase C precursor, chloroplast ferredoxin-dependent glutamate synthase, and heat shock protein (HSP)-interacting protein. All of them were related to drought or adversity stress, and the function of three target genes was annotated as unnamed protein products. The functions of the two target genes were unknown (Table 3).

**Table 3:** Differentially expressed novel miRNA and differentially expressed target genes.

Novel miRNA	Regulation	log2 (fold_change)	*p* Value (t_test)	Accession	Annotation	Regulation	Significant
PC-3p-105150_88	Up	inf	0.04	TRINITY_DN15808_c0_g1	Protein ROOT PRIMORDIUM DEFECTIVE 1-like	Down	Yes
PC-3p-105150_88	Up	inf	0.04	TRINITY_DN19688_c0_g2	Uncharacterized oxidoreductase At1g06690, chloroplastic-like	Down	Yes
PC-3p-68960_194	Up	0.87	0.01	TRINITY_DN14521_c0_g2	Protein MAK16 homolog	Down	Yes
PC-3p-77094_159	Up	inf	0.00	TRINITY_DN11948_c0_g1	Unnamed protein product	Down	Yes
PC-3p-77094_159	Up	inf	0.00	TRINITY_DN12469_c0_g1	Unnamed protein product	Down	Yes
PC-3p-77094_159	Up	inf	0.00	TRINITY_DN14567_c0_g1	Uncharacterized protein LOC109765237	Down	Yes
PC-3p-77094_159	Up	inf	0.00	TRINITY_DN17145_c0_g2	Protein TSS isoform X1	Down	Yes
PC-3p-77094_159	Up	inf	0.00	TRINITY_DN17291_c1_g2	RecName: Full = RuBisCO large subunit-binding protein subunit beta, chloroplastic; AltName: Full = 60 kDa chaperonin subunit beta; AltName: Full = CPN-60 beta	Down	Yes
PC-3p-77094_159	Up	inf	0.00	TRINITY_DN18346_c1_g2	Uncharacterized protein LOC109753311	Down	Yes
PC-3p-77094_159	Up	inf	0.00	TRINITY_DN20477_c0_g1	Magnesium-chelatase subunit CHLH, chloroplastic	Down	Yes
PC-3p-77094_159	Up	inf	0.00	TRINITY_DN22992_c0_g1	Predicted protein	Down	Yes
PC-3p-77094_159	Up	inf	0.00	TRINITY_DN25063_c0_g5	Predicted protein	Down	Yes
PC-3p-77094_159	Up	inf	0.00	TRINITY_DN25165_c0_g3	NADP-thioredoxin reductase C precursor, partial	Down	Yes
PC-3p-77094_159	Up	inf	0.00	TRINITY_DN25174_c0_g2	Elongation factor 2	Down	Yes
PC-3p-77094_159	Up	inf	0.00	TRINITY_DN26970_c0_g2	Chloroplast ferredoxin-dependent glutamate synthase	Down	Yes
PC-3p-77094_159	Up	inf	0.00	TRINITY_DN28818_c0_g1	Heat shock factor A6	Down	Yes
PC-3p-77094_159	Up	inf	0.00	TRINITY_DN28919_c0_g1	Isoleucine--tRNA ligase, chloroplastic/mitochondrial-like isoform X1	Down	Yes
PC-3p-77094_159	Up	inf	0.00	TRINITY_DN3290_c0_g1	HSP-interacting protein	Down	Yes
PC-3p-85874_131	Down	−1.32	0.01	TRINITY_DN17343_c0_g5	-	Up	Yes
PC-3p-85874_131	Down	−1.32	0.01	TRINITY_DN17607_c0_g3	Hypothetical protein TRIUR3_23162	Up	Yes
PC-3p-85874_131	Down	−1.32	0.01	TRINITY_DN21478_c1_g5	Predicted protein	Up	Yes
PC-3p-85874_131	Down	−1.32	0.01	TRINITY_DN26358_c3_g4	VQ motif-containing protein 29-like	Up	Yes
PC-5p-41033_410	Up	1.09	0.05	TRINITY_DN25475_c1_g1	Unnamed protein product	Down	Yes
PC-5p-41033_410	Up	1.09	0.05	TRINITY_DN28714_c0_g2	Phosphomethylethanolamine N-methyltransferase-like	Down	Yes
PC-5p-41033_410	Up	1.31	0.02	TRINITY_DN15316_c2_g6	Chaperone protein ClpD2, chloroplastic	Down	Yes
PC-5p-41033_410	Up	1.31	0.02	TRINITY_DN28714_c0_g2	Phosphomethylethanolamine N-methyltransferase-like	Down	Yes
PC-5p-77163_159	Up	2.24	0.03	TRINITY_DN16050_c0_g1	Acetate/butyrate--CoA ligase AAE7, peroxisomal-like	Down	Yes
PC-5p-77163_159	Up	1.97	0.03	TRINITY_DN16730_c0_g8	Putative ripening-related protein 6	Down	Yes
PC-5p-77163_159	Up	1.97	0.03	TRINITY_DN19390_c0_g2	-	Down	Yes
PC-5p-77163_159	Up	1.97	0.03	TRINITY_DN23892_c0_g5	Inositol-tetrakisphosphate 1-kinase 2-like	Down	Yes
PC-5p-77163_159	Up	1.97	0.03	TRINITY_DN28714_c0_g2	Phosphomethylethanolamine N-methyltransferase-like	Down	Yes
PC-5p-78312_155	Up	inf	0.04	TRINITY_DN14321_c1_g1	Translation factor GUF1 homolog, chloroplastic	Down	Yes
PC-5p-78312_155	Up	inf	0.04	TRINITY_DN15829_c0_g2	hypothetical protein	Down	Yes
PC-5p-78312_155	Up	inf	0.04	TRINITY_DN25857_c0_g2	Hypothetical protein	Down	Yes
PC-5p-78312_155	Up	inf	0.04	TRINITY_DN28023_c0_g4	Luminal-binding protein 2	Down	Yes
PC-5p-87000_128	Up	inf	0.04	TRINITY_DN28714_c0_g2	Phosphomethylethanolamine N-methyltransferase-like	Down	Yes

### Gene co-expression network module construction

**Figure 5:**
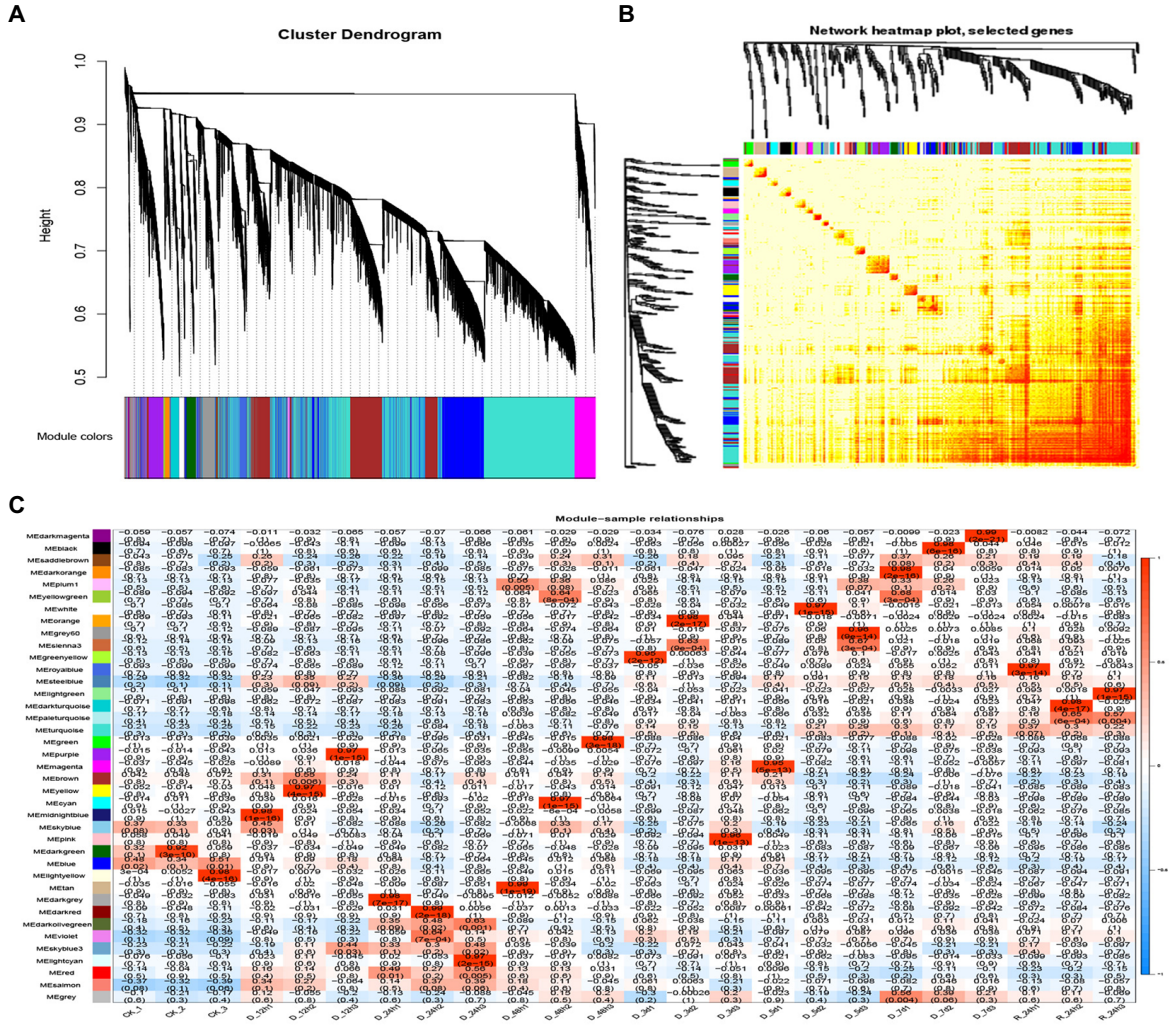
Correlation between sample clustering of the modules and genes. **(A)** Clustering dendrograms of the genes. Based on the dynamic hybrid branch cutting method, color strips were used for simple visualization of the module assignment (branch cutting). **(B)** 41,793 gene and module correlation clustering diagrams. **(C)** Heat map of the correlations between the modules and genes of different samples. The colors that range from blue through white to red indicate low through intermediate to high correlations, respectively.

A WGCNA analysis indicated that genes with similar functions cluster together in the same module (Langfelder and Horvath, 2008). To identify hub genes and their interacting genes of *A. mongolicum* involved in drought resistance, 41,793 genes were used to construct gene co-expression networks and modules, which yielded 39 modules (Figure 5A).The turquoise module was the largest and contained 10,766 genes, while the smallest module was the plum1 module, which included 36 genes (Supplementary Table 12). The module heat map of gene clustering was constructed based on the correlation of expression of feature genes. The gene clusters with highly correlated expression corresponded to a branch of the clustering tree. The clustering heat map drawn by the neighbor relationship was consistent with the clustering results drawn by dynamic shea (Figure 5B). A total of 39 modules of all genes from 24 samples were used to create cluster heatmaps for *A. mongolicum* (Figure 5C). The violet and darkolivegreen modules were highly correlated with drought-treated samples at D_24 h2 and D_24 h3. The red and salmon modules positively correlated with drought-treated samples at D_24 h3; and the turquoise module positively correlated with the drought-treated samples at 5 d, 7 d, and R_24 h. The steelblue module was positively associated with drought-treated samples at 12 h, 5 days, 7 days, and R_24 h. The modules involved in drought stress were initially screened by a module-trait interrelationship heatmap.

### Analysis of the drought-related modules

**Figure 6:**
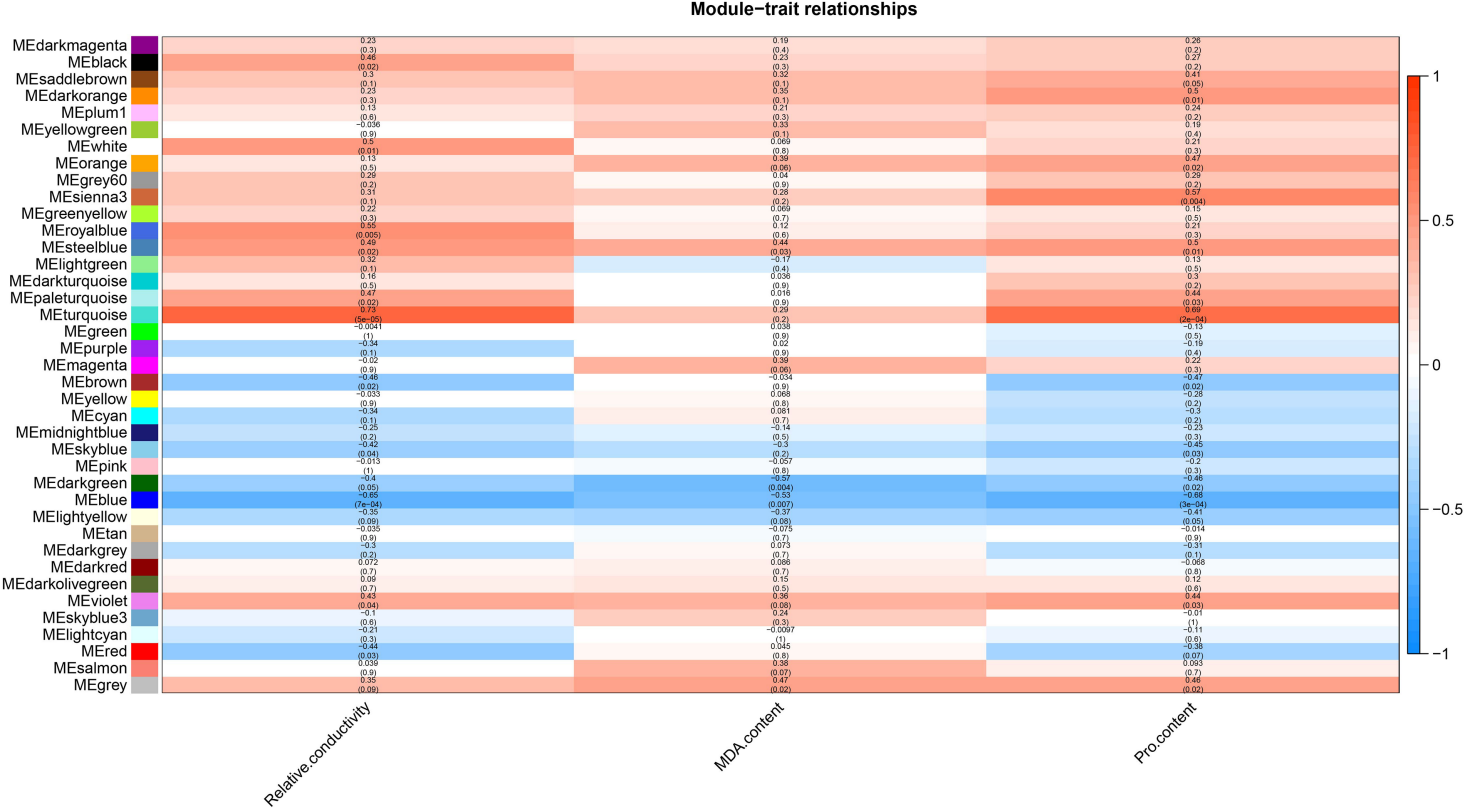
Heat map of the correlation between modules and traits.

Relative conductivity, proline (Pro) and malondialdehyde (MDA) contents are closely related to the drought tolerance of plants. The use of module-trait correlation analysis facilitates the study of complex drought effects. The relative conductivity, Pro and MDA contents of *A. mongolicum* were measured under 25% PEG-6000 treatment (Supplementary Figure 3). In *A. mongolicum*, a heat map of correlations between the module and trait was created using 39 modules and physiological indices of drought resistance, such as relative conductivity and the contents of MDA and Pro (Figure 6). A correlation analysis showed that the turquoise module positively correlated with relative conductivity and Pro, with correlation coefficients of 0.73 (*p* = 5e^−05^) and 0.69 (*p* = 2e^−04^), respectively, and the correlation coefficient of MDA was 0.29 (*p =* 0.2). The steelblue module significantly and positively correlated with relative conductivity, MDA, and Pro, with correlation coefficients of 0.49 (*p =* 0.02), 0.44 (*p =* 0.03), and 0.5 (*p =* 0.01), respectively. The violet module positively correlated with relative conductivity and Pro with correlation coefficients of 0.43 (*p =* 0.04) and 0.44 (*p =* 0.03), respectively, and the correlation coefficient of MDA was 0.36 (*p =* 0.08). The blue module significantly and negatively correlated with relative conductivity, MDA, and Pro with correlation coefficients of −0.65 (*p =* 7e^−04^), −0.53 (*p =* 0.007), and −0.68 (*p =* 3e^−04^), respectively.

### GO and pathway analysis of genes in specific modules

**Figure 7:**
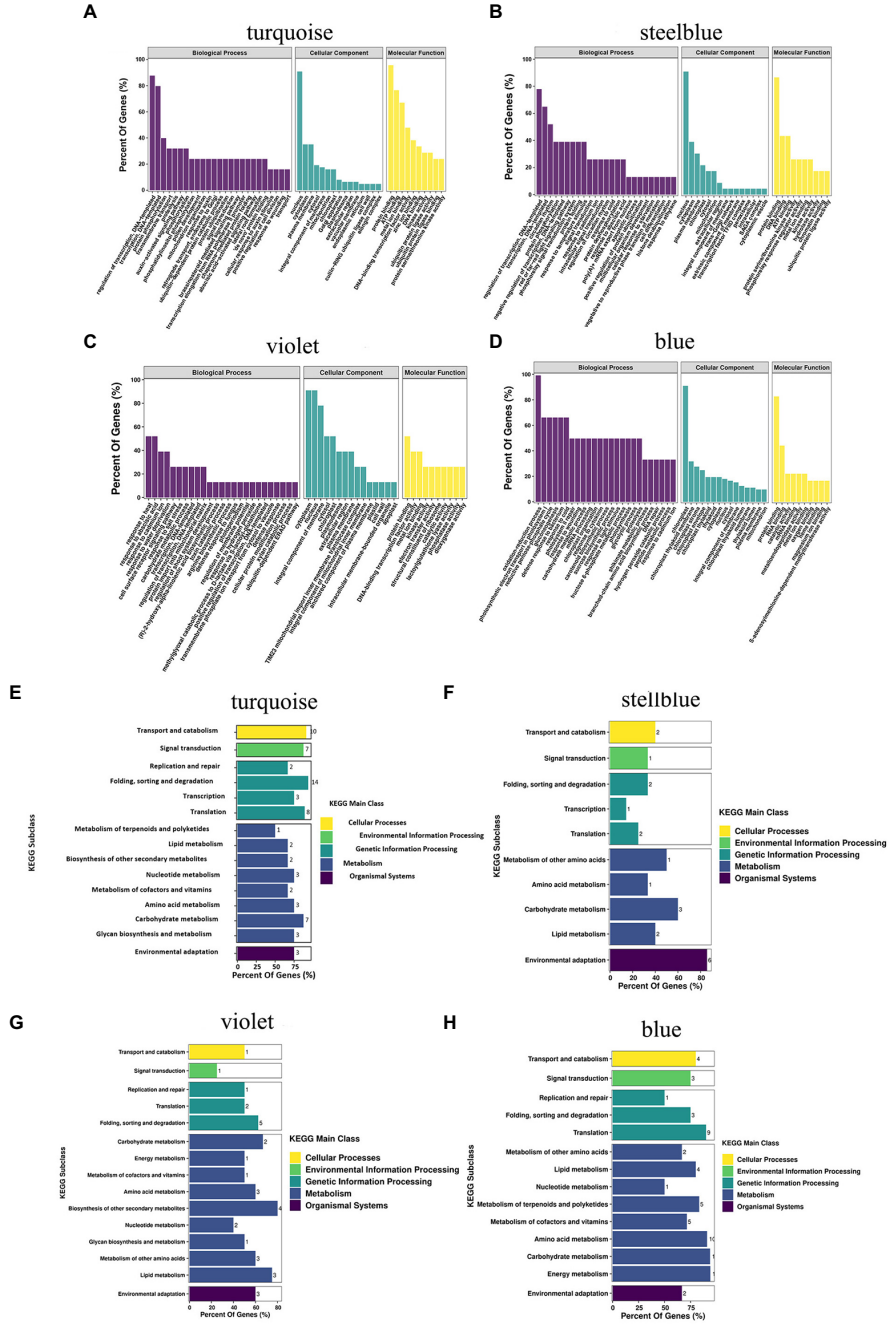
GO classification and putative KEGG pathways of specific modules related to drought resistance in *Agropyron mongolicum.* The GO classification and KEGG pathway of turquoise, steelblue, violet, and blue modules are represented by **(A)**, **(B)**, **(C)**, **(D)**, **(E)**, **(F)**, **(G)**, and **(H)**, respectively. GO, Gene Ontology; KEGG, Kyoto Encyclopedia of Genes and Genomes.

Under drought treatment, the GO and KEGG analyses showed the functions and pathways of genes in specific modules of *A. mongolicum*. Biological processes, molecular functions, and cellular components were enriched in the GO analysis of turquoise, steelblue, violet, and blue modules (Figures 7A–D). In biological processes, it was primarily enriched for the regulation of transcription, DNA-templated (GO:0006355), protein phosphorylation (GO:0006468), signal transduction (GO:0009737), brassinosteroid-mediated signaling pathway (GO:0009742), abscisic acid (ABA)−activated signaling pathway (GO:0009737, GO:0009788, GO:0009737, GO:0009738), cell surface receptor signaling pathway (GO:0007166), positive regulation of protein ubiquitination (GO:0031398), and response to water deprivation (GO:0009414) processes. The biological processes played an essential role in the mechanism of *A. mongolicum* related to drought resistance. Cellular Component analysis revealed that the four modules were primarily enriched in the nucleus (GO:0005634), plasma membrane (GO:0006351, GO:0005886, and GO:0005829), cytoplasm (GO:0005737), cytosol and chloroplast (GO:0016021 and GO:0009941) components. The results showed that cellular components, such as the nucleus, cytoplasm, plasma membrane and chloroplast, responded to drought stress. Molecular function was primarily enriched to protein binding (GO:0005515), metal ion binding (GO:0046872, GO:0008270), kinase activity (GO:0016301) and other functions, which could play an important role in drought regulation in *A. mongolicum*. The KEGG pathway from four modules of *A. mongolicum* was primarily enriched for transport and catabolism under drought stress, signal transduction, amino acid metabolism, carbohydrate metabolism, and environmental adaptation, indicating that these pathways responded to stimulation in response to drought (Figures 7E–H).

### Analysis of drought-resistant hub genes

**Figure 8:**
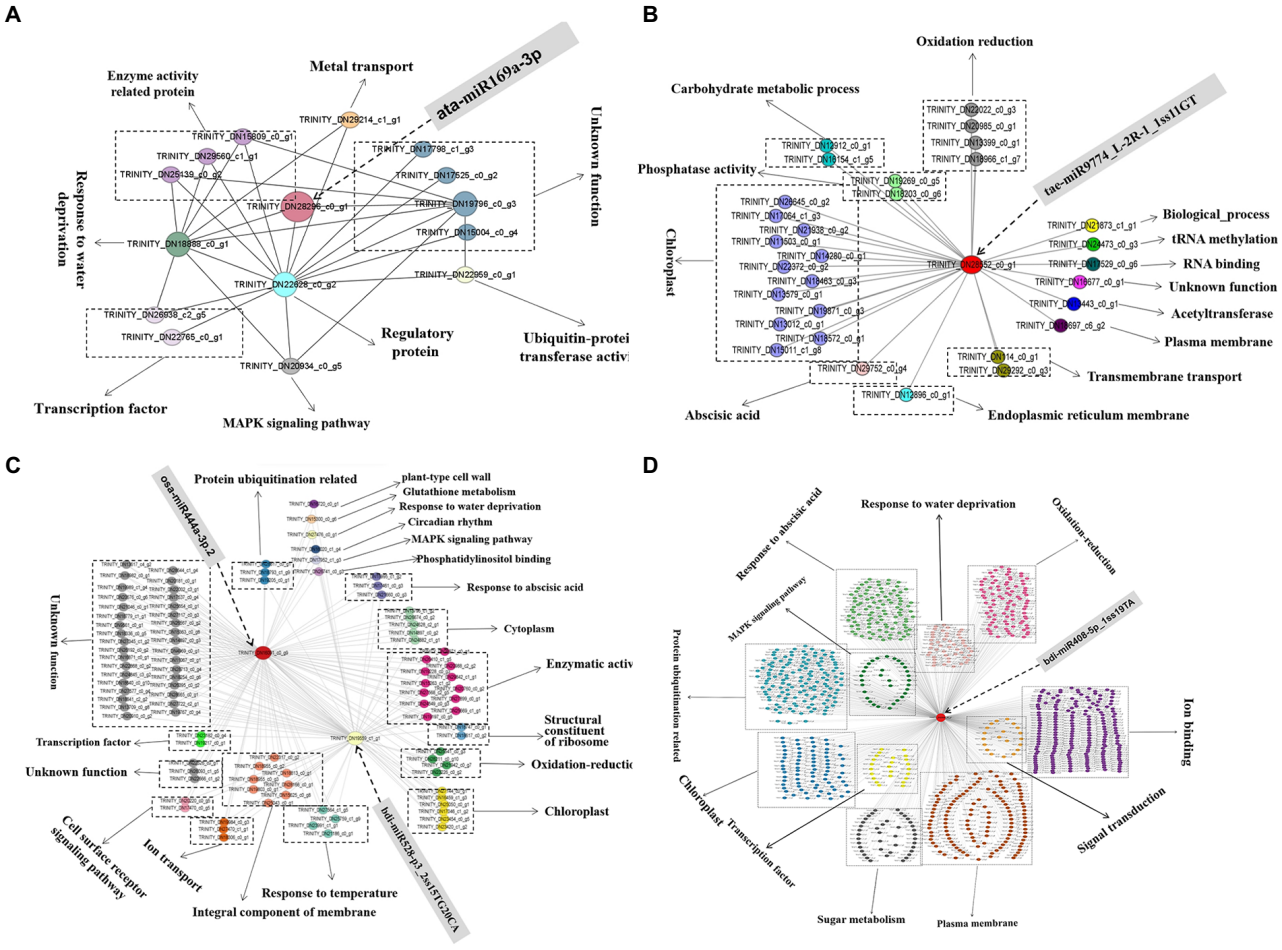
The coexpression subnetwork of five hub genes. Red-colored nodes suggest their central role in the network, and other nodes of the same color indicate the same function. **(A)**, **(B)**, **(C)**, and **(D)** represent the coexpression subnetwork of TRINITY_DN28296_c0_g1 (*PAO2*), TRINITY_ DN28552_c0_g1 (*carC*), TRINITY_DN16091_c0_g9 (MADS47), TRINITY_DN19559_c1 _g1 (*HOX24*), and TRINITY_DN29080_c0_g1 (*CCX1*), respectively. A part of the gene ID corresponds to the gene name: *Os06g0107700* (TRINITY_DN22022_c0_g3), *XB3* (TRINITY_DN22959_c0_g1), *CLPC1* (TRINITY_DN21467_c0_g3), and *BPM2* (TRINITY_DN19691_c0_g7).

The top 20 genes in the four modules related to the drought tolerance of *A. mongolicum* with connectivity were used as hub genes, and the genes that interacted with the hub genes were selected (Supplementary Table 12). The differentially expressed miRNAs with a targeted relationship to the hub genes were screened based on the degradome and miRNA sequencing data. The five hub genes that targeted/regulated differentially expressed miRNAs were analyzed, including *MADS47* (TRINITY_DN16091_c0_g9, GO Function annotation: brassinosteroid mediated signaling pathway) targeted/regulated osa-miR444a-3p.2 in the violet module (*p* < 0.01), *CCX_1_* (TRINITY_DN29080_c0_g1, GO Function annotation: sodium and potassium ion transport) targeted/regulated bdi-miR408-5p_1ss19TA in the turquoise module (*p* < 0.05), steelblue module *PAO2* (TRINITY_DN28296_c0_g1, GO Function annotation: oxidation–reduction process, peroxisome) targeted/regulated ata-miR169a-3p (*p* < 0.01) and *carC* (TRINITY_DN28552_c0_g1, GO Function annotation: hydrolase activity) targeted/regulated tae-miR9774_L-2R-1_1ss11GT in the blue module (*p* < 0.01). The *HOX24* (TRINITY_DN19559_c1_g1) targeted/regulated bdi-miR528-p3_2ss15TG20CA was found in the violet module, and the GO term of the *HOX24* gene was annotated as a signaling pathway in the response to water deprivation and in response to ABA and the ABA−activated signaling pathway, indicating that *HOX24* may play an important role in drought stress resistance. The regulatory network and function of hub genes and interacting genes were deeply explored to better understand the mechanism of drought resistance in *A. mongolicum* (Figure 8). When *A. mongolicum* was subjected to drought stress, *PAO2* controlled the functional genes of water deficit and regulatory proteins (TRINITY_DN18888_c0_g1, TRINITY_DN22628_c0_g2, and TRINITY_DN19796_c0_g3), suggesting that *PAO2* may indirectly controls the enzyme activity-related proteins, ubiquitin-protein transferase activity, MAPK signaling pathway, and other functional genes. This indicates that *PAO2* may play a key role in the gene coexpression regulatory network. *carC* would activate many of the genes that regulate chloroplast functions in response to drought stress and then regulate redox, carbohydrate metabolism, and phosphatase activity. *MADS47* and *HOX24* are the hub genes in the violet module with the connectivity of top1 and top15. These two genes may have direct interactions and co-regulate the downstream functional genes of kinase activity, oxidation–reduction and chloroplast. Secondly, the genes that govern enzyme activity, chloroplast, cytoplasm, protein ubiquitination, and cell components function are activated to achieve drought regulation. *MADS47* is presumed to be the master gene in this module based on gene connectivity. There were 7,993 genes with interactions with *CCX1*, and 905 of the genes were chosen for functions related to water deficiency response, MAPK signaling pathway, and protein ubiquitination, including 204 genes for ion binding, transport, and exchange; 138 genes for protein ubiquitination; 122 genes for membrane function; and 102 genes for phytohormones, followed by redox regulation (90), chloroplast (82), water deficiency response (49), sugar (42). *CCX1* of *A. mongolicum* would activate the interacting genes under drought stress, which mobilizes drought defense mechanisms to manage drought. The regulatory networks provide important references for subsequent studies of drought resistance mechanisms in *A. mongolicum*.

### Correlation analysis of miRNAs and their candidate hub genes for drought resistance

**Figure 9:**
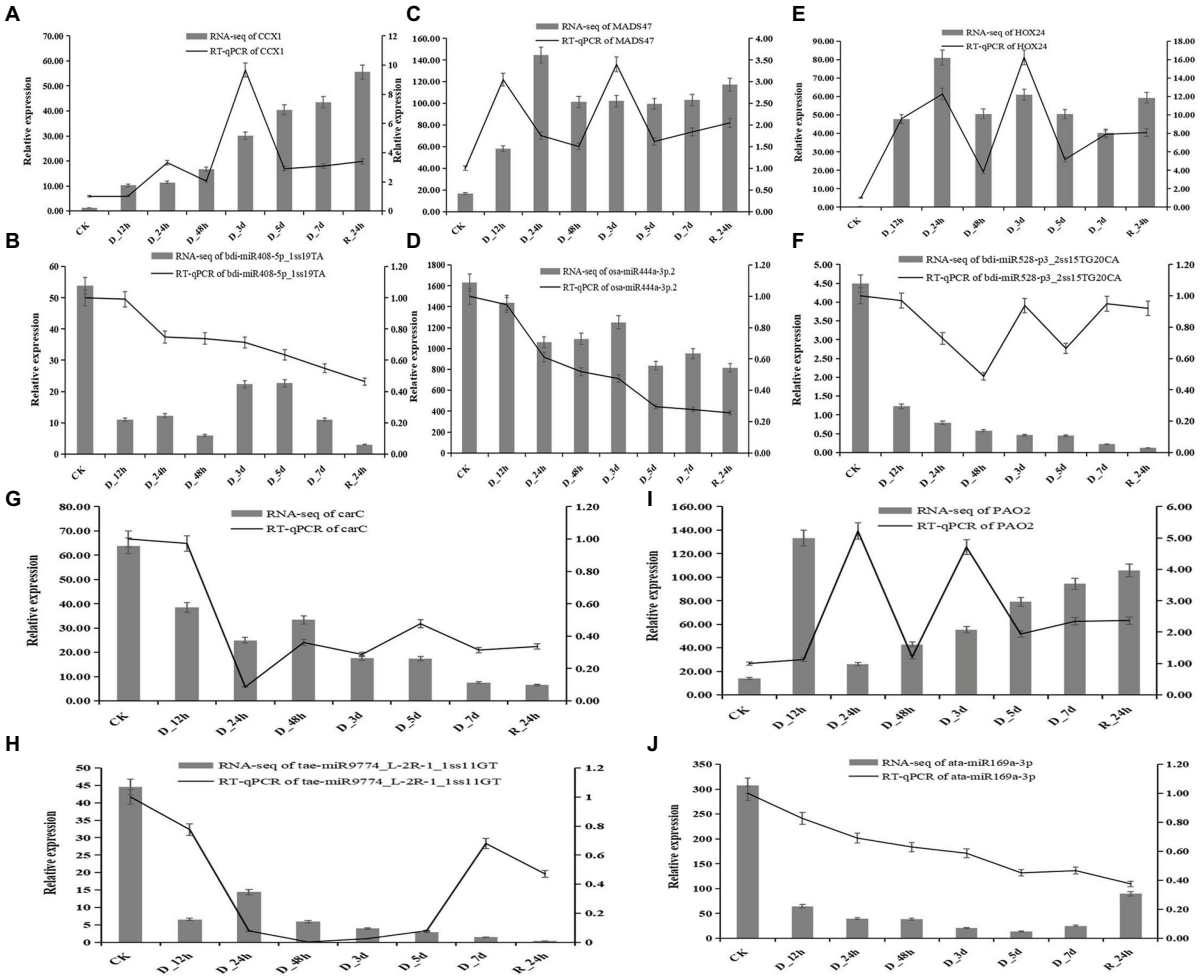
Correlation of the expression between miRNAs and the hub genes at eight durations of different drought treatments (25% PEG solution PEG6000). bdi-miR408-5p_1ss19TA target *CCX1* **(A,B)**; osa-miR444a-3p.2 target *MADS47* **(C,D)**; bdi-miR528-p3_2ss15TG20CA target *HOX24* **(E,F)**; tae-miR9774_L-2R-1_1ss11GT target carC **(G,H)**; and ata-miR169a-3p target *PAO2* **(I,J)**.

Five miRNAs and their target genes were chosen for RT-qPCR to better understand the connection between the expression of miRNA and its drought-resistant potential hub genes (Figure 9). The patterns of expression of four miRNA-target gene pairings (bid-miR408-5p_1ss19TA-*CCX1*, ata-miR169a-3p-*PAO2*, osa-miR444a-3p.2-*MADS47*, and bdi-miR528-p3_2ss15TG20CA-*HOX24*) were all negatively regulated. The levels of expression of four miRNAs decreased as the drought treatment period increased, but the relative expression of their target genes increased. The levels of expression of target the genes increased after 24 h of rehydration. The relative expression of tae-miR9774_ L-2R-1_1ss11GT and target gene carC decreased overall.

## Discussion

*Agropyron mongolicum* is a vital forage grass and has exceptional drought and cold resistance. It is grown in semiarid and arid desert regions where it is subjected to abiotic stresses, particularly drought stress. *A. mongolicum* has developed a robust set of mature regulatory mechanisms to manage the response to drought stress. The severity of drought is determined by various factors, such as rainfall, the ability of soils to store moisture, and evaporation demand. Drought has many effects on crops and can deleteriously affect metabolism, photosynthesis and defense mechanisms (Andrew et al., 2009). Plants respond to drought through root development, stomatal opening and closing, cell adaptation, ABA and reactive oxygen species (ROS) scavenging (Abid et al., 2017). The genes of exceptional drought resistance in *A. mongolicum* could be beneficial to improving the drought tolerance of gramineous crops, such as wheat and rice. Therefore, it is imperative to study the molecular mechanism of drought resistance in *A. mongolicum*.

Drought stress has been demonstrated to induce the particular expression of drought-related genes in plants (Bano et al., 2022). Three important high-throughput methods, the transcriptome, small RNA, and degradome sequencing, were applied to investigate the mechanism of drought resistance in *A. mongolicum*. In comparison with the miRNAs identified from the other plants, little research has been conducted on the role of miRNAs in *A. mongolicum*. To date, 114 miRNAs that are related to the drought resistance of *A. mongolicum* have been reported in our previous research group, and the functions of amo-miR21, amo-miR5 and amo-miR623 have been correlated with drought resistance (Zhang et al., 2019). The transcriptome dataset of *A. mongolicum* was used as a reference sequence for small RNA and degradome sequencing analyses to identify the miRNAs and their target genes that could be associated with drought stress. A total of 1,104 miRNAs, including 101 highly confident miRNAs (Supplementary Table 7), were identified based on the transcriptome data from *A. mongolicum*. Additionally, the miRNA lengths ranged from 18 to 25 nt, with a peak at 24 nt (Supplementary Figures 2C,D).

The results of the sRNA molecules of *A. mongolicum* match those of most plant miRNAs from prior studies (Zhao et al., 2010a; Niu et al., 2014; Bin et al., 2021). Some miRNAs that responded to drought in previous studies were also detected in our results, including miR156, miR159, miR162, miR171, miR444, and miR408. For example, rice tandem osa-miR156b and osa-miR156c were overexpressed in alfalfa (*Medicago sativa*). The expression of miR156 increased significantly in transgenic alfalfa, and the plants that overexpressed the gene were better able to tolerate salt and drought resistance (Wang et al., 2021b). The overexpression of miR171f in rice resulted in reduced drought symptoms in transgenic plants compared with the control plants, which were found to play a role in drought tolerance by targeting SCL6-I and SCL6-II (Taeyoung et al., 2022). Sly-miR159 targets S*lMYB33* to regulate the accumulation of proline and putrescine to improve drought resistance (López et al., 2019). The data described above indicate that the sRNA sequencing results of *A. mongolicum* under drought stress are authentic and reliable.

In the WGCNA analysis, the unearthed potential drought-resistant hub genes (*MADS47*, *CCX1*, *carC*, *PAO2*, and *HOX24*) targeted five miRNAs, which were osa-miR444a-3p.2, bdi-miR408-5p_1ss19TA, tae-miR9774_L-2R-1_1ss11GT, ata-miR169a-3p, and bdi-miR528-p3_2ss15TG20CA. Further study of the function of these miRNA targets provides valuable information about the regulatory pathways available for any given gene. The bdi-miR528-p3_2ss15TG20CA expression of *HOX24* may activated the ABA−activated signaling pathway. osa-miR444a-3p.2 was differentially expressed in *A. mongolicum* under drought stress (*p* ≤ 0.001) and predicted to regulate *MADS47* target genes. The regulation of *MADS47* by osa-miR444a-3p.2a may affect the brassinosteroid-mediated signaling pathway. Furthermore, the redox process may regulate by ata-miR169a-3p and miR9774, and sodium and potassium ion transport may regulate by bdi-miR408-5p_1ss19TA.

miR444 is specific to monocots and has been confirmed to target four MIKC-type MADS-box genes in rice (*OsMADS23*, *OsMADS27a*, *OsMADS27b*, and *OsMADS57*) (Sunkar et al., 2005; Lu et al., 2008; Wu et al., 2009; Li et al., 2010). Recent research provides evidence of a nitrate-dependent miR444-*OsMADS27* signaling cascade involved in the regulation of root growth in rice, as well as its dramatic role in stress responses (Kannan et al., 2022). The overexpression of miR444 promotes the biosynthesis of brassinosteroids (BRs) and inhibits the elongation of roots in rice (Jiao et al., 2020). In previous research, few studies have reported on miR9774, which is found in the mixed tissues of wheat leaves, stems, roots, and young spikes (Wei et al., 2009). In the low Cd-accumulation of wheat (Chuanyu 17), tae-miR-9,774 downregulated the expression between the group treated with 100 μM CdCl_2_ and the control group (Zhou et al., 2020). miR408, a conserved and ancient miRNA found widely in plants, is involved in photosynthesis and controls the target genes of copper-binding proteins (Gao et al., 2022), which is vital for leaf growth. miR408 has been linked to drought and water shortage stress in *A. thaliana*, rice (Roseeta et al., 2013; Sonia et al., 2017), wheat (Guray et al., 2016), tomato (*Lycopersicon esculentum*; Bilgin et al., 2016), and perennial ryegrass (*Lolium perenne*; Nan et al., 2020). The antioxidant capability of plants can be improved by miR408, which enhances cellular antioxidants (Gao et al., 2022). In chickpea (*Cicer arietinum*), the expression of *PLC* (a target of miR408) decreased significantly with the overexpression of miR408 to provide drought tolerance (Hajyzadeh et al., 2015). When *A. thaliana* was exposed to drought stress, miR408 was upregulated, while its target genes *PLC* and *LAC3* were downregulated (Chao et al., 2015). miR169 was identified for its involvement in drought stress in *A. thaliana* (Du et al., 2017b), poplar (*Populus* spp.), rape (*Brassica napus*), and potato (*Solanum tuberosum*; Yang et al., 2016) crops. For example, in response to drought and ABA treatment, Bna-miR169n targets *BnaNF-YA9* in *Brassica napus* (Wang et al., 2022). PtmiR169o targets the *PtNF-YA6* gene to regulate drought tolerance in poplar (Jiao et al., 2021). More research into miR169-targeted *PAO2*-regulated drought is required. miR528 is involved in biotic stresses in plants, such as drought tolerance and salt tolerance stress. In rice, the overexpression of *TCONS_00021861* attenuated the repression of miR528-3p on *YUCCA7*, which, in turn, activated the indole acetic acid (IAA) biosynthetic pathway and conferred resistance to drought stress (Chen et al., 2021). Durum wheat ata-miR528-5p promotes redox homeostasis by targeting the F-box protein and Cu Zn superoxide dismutase (Cu Zn SOD) under stress (Liu et al., 2020). These findings suggest that the known miRNAs are likely to be engaged in cross-adaptation to control plant tolerance to abiotic conditions, such as drought. However, their specific duties and exact functions need to be verified in the future.

It is impossible to extrapolate the complete picture of a complex environmental component, such as drought regulation, from the effects of individual genes. A combination of transcriptome, degradome and miRNAs expression profiles is needed to elucidate the synergistic impact of drought-related genes (Liu et al., 2020). A total of 41,792 UniGenes (Table 2) were detected in the *A. mongolicum* transcriptome. Under specific abiotic stresses, such as drought stress, mRNAs have a particular expression in response to the pressure. Thus, there were 1,166 DEGs in the seven comparison groups under drought stress (Figure 2). The number of up- and downregulated genes also differed among the various comparison groups. The level of gene expression could differ owing to the varying drought treatment times. GO classification to drought-related GO terms include response to salt stress, response to ABA and response to wounding (Figure 1A). The degradome data identified 137 *A. mongolicum*-specific miRNAs that target 1,394 genes (Supplementary Table 9). The integrated analysis of transcriptome, sRNAs and degradome that focused on miRNAs and their target genes in negative regulatory mode, identified 91 miRNAs that negatively regulate 1,438 target genes. Some DEGs were commonly up- or downregulated under drought stress, while others exhibited unique patterns. In *A. mongolicum*, we observed that eight novel miRNAs with different expression patterns regulated 36 target genes. The 36 target genes that are controlled (Figure 4; Supplementary Table 10) were annotated under drought stress as uncharacterized oxidoreductase, magnesium-chelatase subunit CHLH chloroplastic, NADP-thioredoxin reductase C precursor, chloroplast ferredoxin-dependent glutamate synthase, and heat shock protein-interacting protein (Table 3). This study showed that all these functional annotations are involved in drought stress regulation. For example, the Chloroplastic, Magnesium Protoporphyrin-IX Chelatase H subunit (CHLH) receptor has been found to bind ABA, and CHLH is a crucial enzyme in chlorophyll production (Wasilewska et al., 2008; Zhang et al., 2019). NADP has been shown to regulate the maintenance of redox homeostasis and respond to environmental stresses in plants (Wang et al., 2020). According to the findings, drought stress is regulated in plants by a variety of pathways, including chlorophyll and ABA regulation. Plants that respond to drought stress could also activate pathways to other stresses, such as salt stress or heat stress, or some of the pathways could be shared by plants that respond to biotic stresses.

To identify the highly putative genes in drought responses in *A. mongolicum*, 41,792 mRNAs were analyzed using WGCNA technology based on transcriptome sequencing data to study the hub genes and co-expression networks in response to drought stress. A total of 39 co-expression modules were constructed, and four modules related to drought resistance were enriched for signal transduction, brassinosteroid mediated signaling pathway, and the ABA−activated signaling pathway (Figure 7). These enriched functions were closely related to drought stress. *MADS47*, *CCX1*, *carC*, *PAO2*, and *HOX24* were five hub genes with putative drought tolerance (Figure 8). *MADS47* is a MADS transcription factor, which plays essential functions in plant growth and development and response to adversity stress (Natalia et al., 2019; Zhao et al., 2020; Han et al., 2022a). *OsMADS2*, *OsMADS30*, and *OsMADS55* were downregulated in expression under drought and salt stress in rice (Rita et al., 2007). However, the RT-qPCR results of *A. mongolicum* showed that the relative level of expression of *MADS47* was upregulated in drought stress. In rice, *OsMADS26* plays a negative regulatory role and reduces the drought resistance of the plant, which is the same regulatory pattern as *MADS47* in *A. mongolicum*. *MADS47* is a hub gene in the regulatory network and is functionally shown to be associated with the BR-mediated signaling pathway. BRs are plant hormones that promote growth, improve plant stress resistance, and regulate the level of expression of chlorophyll synthase genes (Ryo et al., 2022). BR can promote wound healing by activating reactive oxygen metabolism (Han et al., 2022b).

The *HOX24* annotation information is related to ABA and water deficit regulation. A Homeo-Leucine Zipper (HD-Zip) is a class of transcription factors that is unique to higher plants and involved in stress response and the growth and development of plants. *ATHB-6* is the HD-zip gene of maize (*Zea mays*), and the overexpression of *ATHB-6* in maize activates the expression of critical genes in the ROS signaling pathway and ABA-dependent pathway under drought tolerance (Peng et al., 2022). The annotated function of *HOX24* in sugarcane (*Saccharum officinarum*) under drought stress is related to antioxidants (Peiting et al., 2022). Both *MADS47* and *HOX24* in *A. mongolicum* are hub genes in the violet module, and they are mutually regulated and have genes that act together. Their functions are annotated as enzymatic activity, oxidation–reduction and cell surface receptor signaling pathway. The ABA hormone and BRs are the primary regulatory molecules that respond to drought signals and convey them down to the reciprocal genes that they regulate in the *MADS47-* and *HOX24-*mediated regulatory network, after which they exert drought regulation in the form of a network.

*CCX1* is a member of the Cation/Ca^2+^ Exchanger family. The overexpression of *CCX1* in *A. thaliana* increased the sensitivity to hydrogen peroxide (H_2_O_2_) treatment, suggesting that this gene is involved in ROS homeostasis (Li et al., 2016). Na^+^, K^+^, and Ca^2+^ can retard the senescence of broccoli (*Brassica oleracea* var. *italica*) buds. The *CCX1* promoter was cloned in broccoli and found to inhibit its senescence. The functional annotation of *CCX1* in *A. mongolicum* under drought stress is sodium-potassium ion transport (Yan et al., 2020). Previous studies have confirmed the regulatory function of *CCX1* from the side. In the co-expression regulatory network of *CCX1*, which regulates genes with functions in oxidation–reduction and ion binding, protein ubiquitination regulation interacts synergistically in response to drought stress.

Polyamines (PA) are key components of plant growth and development and abiotic stress responses (EAlcázar and Tiburcio, 2014; Wimalasekera et al., 2015). Amine oxidases oxidatively catabolize PAs, and polyamine (PAO) oxidase is one of the breakdown products (Angelini et al., 2010). *PAO2* is annotated as regulating the redox process and peroxisome in *A. mongolicum.* In *A. thaliana*, *AtPAO2* is differentially expressed in the interaction of ABA, nitrate and ammonium ions, which can potentially improve the ability to regulate the growth of *A. thaliana* roots (Wimalasekera et al., 2015). *carC* is a regulatory gene and could play an active role in the production of carotene (Revuelta and Eslava, 1983). *CarC* is annotated as hydrolase activity in *A. mongolicum*. The *carC* coexpression network revealed that it primarily regulates chloroplast and redox genes. There are few reports about the role of *carC* gene in plants. As a result, more research is needed on the mechanism of the drought-resistant regulation of the *carC* gene in *A. mongolicum*. In conclusion, these studies show that *MADS47*, *CCX1*, *carC*, *PAO2*, and *HOX24* genes in drought may play an essential role in *A. mongolicum* under drought stress and can be used as suitable hub genes for drought resistance in *A. mongolicum*.

## Conclusion

This study provides the first integrated analysis of *A. mongolicum* miRNAs and mRNAs regulated at the transcriptional level in response to drought stress. The multi-layered stress-responsive networks mediated by miRNAs and their hub target genes are no doubt highly complex but are coordinated. Five potential drought tolerance hub genes (*MADS47*, *CCX1*, *carC*, *PAO2*, and *HOX24*) were targeted by the analysis and the regulatory network was mapped. The study provides a theory for the functional validation of drought resistance hub genes in *A. mongolicum*. At the same time, new findings would lay the foundations for improving drought tolerance in gramineous crops *via* molecular breeding strategies.

## Data availability statement

The original contributions presented in the study are publicly available. This data can be found at: NCBI, PRJNA742257.

## Author contributions

BF conducted the experiments. YM organized and supervised the overall project. BF, FS, ZY, XZ, XY, JW, XY, LN, and YF performed the editing of the manuscript. YZ provided the seeds of *A. mongolicum*. All authors contributed to the article and approved the submitted version.

## Funding

This study was funded by the National Natural Science Foundation of China (No. 31860670).

## Conflict of interest

The authors declare that the research was conducted in the absence of any commercial or financial relationships that could be construed as a potential conflict of interest.

## Publisher’s note

All claims expressed in this article are solely those of the authors and do not necessarily represent those of their affiliated organizations, or those of the publisher, the editors and the reviewers. Any product that may be evaluated in this article, or claim that may be made by its manufacturer, is not guaranteed or endorsed by the publisher.

## Footnotes

^1^http://www.bioinformatics.babraham.ac.uk/projects/fastqc/ (Accessed August 6, 2022).

^2^https://github.com/trinityrnaseq/trinityrnaseq/wiki (Accessed August 6, 2022).

^3^http://www.ncbi.nlm.nih.gov/ (Accessed August 6, 2022).

^4^http://www.geneontology.org (Accessed August 6, 2022).

^5^http://www.expasy.ch/sprot/ (Accessed August 6, 2022).

^6^http://www.genome.jp/kegg/ (Accessed August 6, 2022).

^7^http://eggnogdb.embl.de/ (Accessed August 6, 2022).

^8^https://horvath.genetics.ucla.edu/coexpression network/ (Accessed August 6, 2022).
