# Corrigendum: Integrated analysis of small RNAs, transcriptome and degradome sequencing reveal the drought stress network in *Agropyron mongolicum* Keng

**DOI:** 10.3389/fpls.2023.1152603

**Published:** 2023-02-08

**Authors:** Bobo Fan, Fengcheng Sun, Zhuo Yu, Xuefeng Zhang, Xiaoxia Yu, Jing Wu, Xiuxiu Yan, Yan Zhao, Lizhen Nie, Yongyu Fang, Yanhong Ma

**Affiliations:** ^1^Agricultural College, Inner Mongolia Agricultural University, Hohhot, China; ^2^Inner Mongolia Academy of Agricultural & Animal Husbandry Sciences, Hohhot, China; ^3^College of Grassland, Resources and Environment, Inner Mongolia Agricultural University, Hohhot, China

**Keywords:** *Agropyron mongolicum* Keng, drought resistance, microRNAs, transcriptome, degradome, integration analysis, co-expression network

In the published article, there was an error in **Figure 9**. **Figure 9** should have been a bar graph of RT-qPCR, but since the relative expression trends of RT-qPCR are the same to that of RNA-seq, we unintentionally put the bar graph of gene expression from RNA-seq in **Figure 9**. We have corrected **Figure 9** to a combined graph from the gene expression of RT-qPCR and RNA-seq.

**Figure 9:**
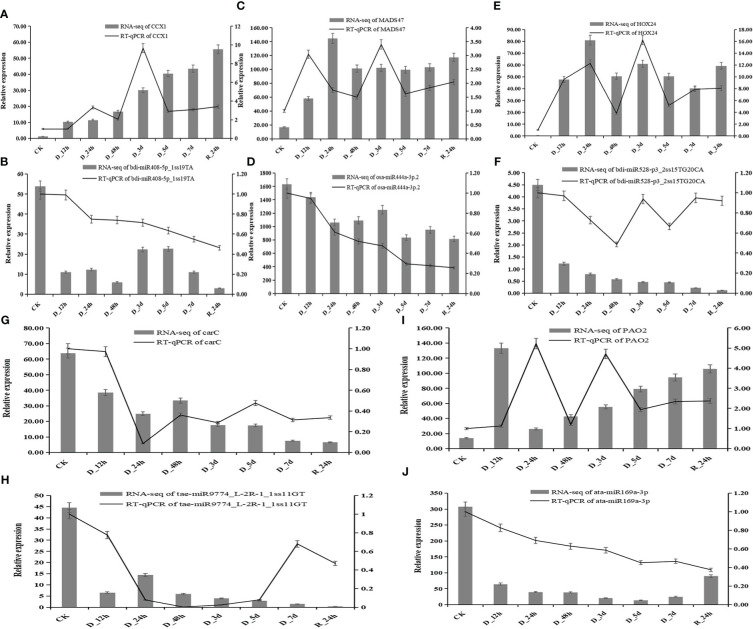
Correlation of the expression between miRNAs and the hub genes at eight durations of different drought treatments (25% PEG solution PEG6000). bdi-miR408-5p_1ss19TA target *CCX1***(A, B)**; osa-miR444a-3p.2 target *MADS47* **(C, D)**; bdi-miR528-p3_2ss15TG20CA target *HOX24* **(E, F)**; tae-miR9774_L-2R-1_1ss11GT target *carC* **(G, H)**; and ata-miR169a-3p target *PAO2* **(I, J)**.

In the last sentence of “Correlation analysis of miRNAs and their candidate hub genes for drought resistance”, “The relative expression of tae-miR9774_ L-2R-1_ 1ss11GT increased overall, but the relative expression of its target genes decreased”, the first “increased” should be “decreased”, and “but the relative expression of its target genes decreased” should be removed.

The corrected sentence appears below:

“The relative expression of tae-miR9774_ L-2R-1_ 1ss11GT and target gene *carC* decreased overall”.

The authors apologize for these errors and state that this does not change the scientific conclusions of the article in any way. The original article has been updated.

