# Supplementary figures and images for: Integrated analysis of small RNAs, transcriptome and degradome sequencing reveal the drought stress network in Agropyron mongolicum Keng
Source: Front Plant Sci. 2022 Aug 17;13:976684. doi: 10.3389/fpls.2022.976684 (PMC9433978; doi:10.3389/fpls.2022.976684)

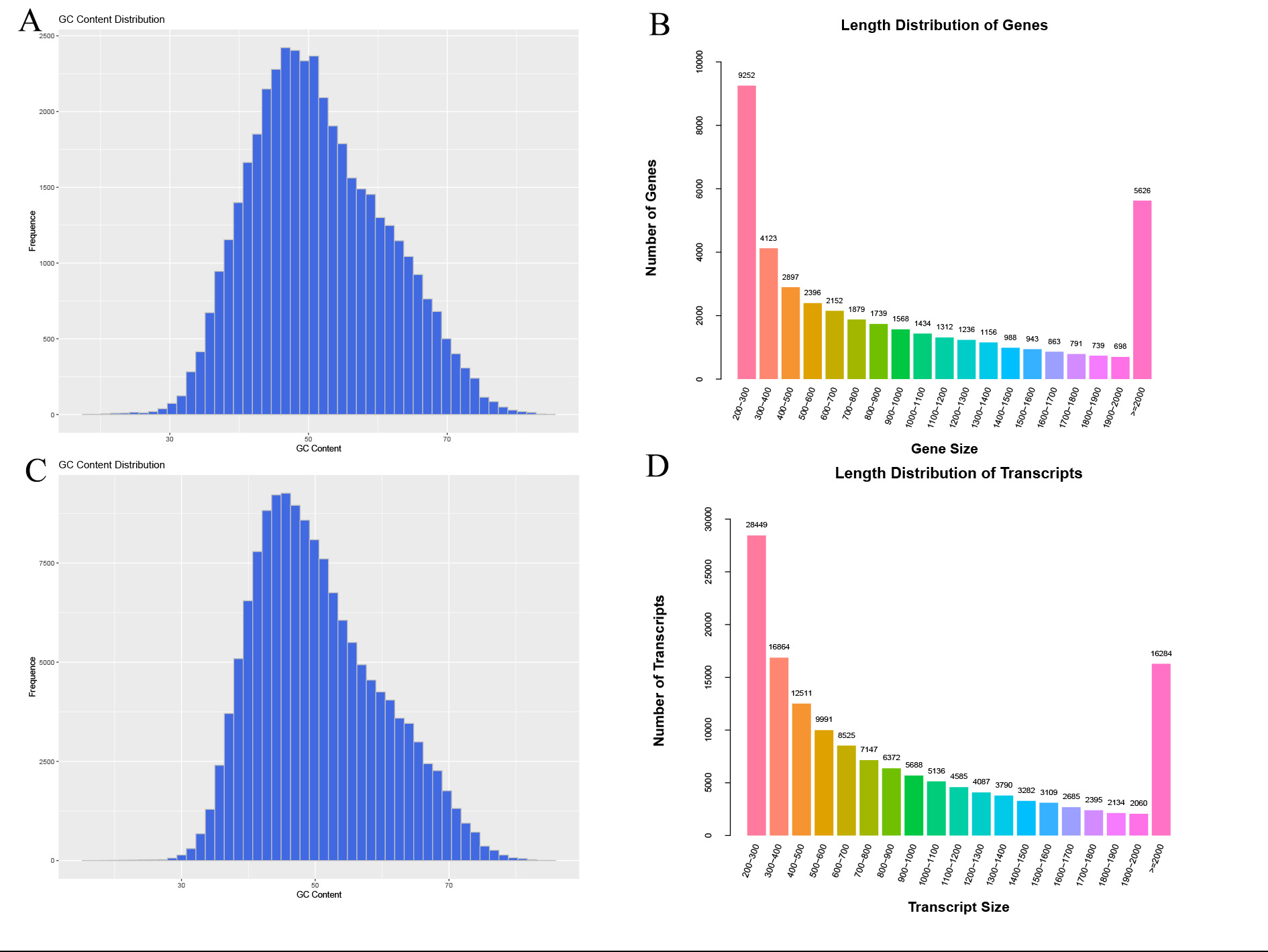

Supplement: Supplementary file 3 [file Image_1.jpg]

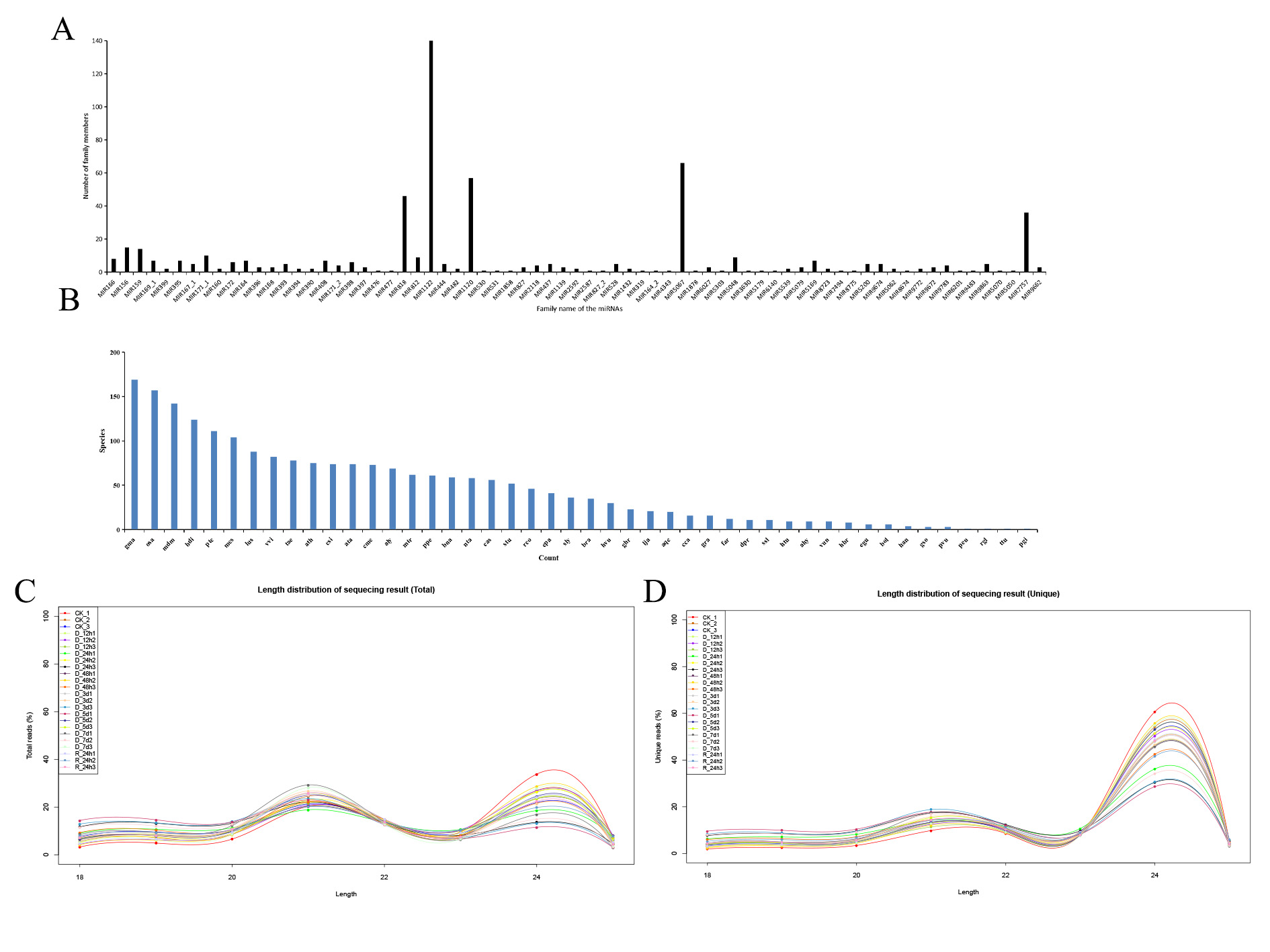

Supplement: Supplementary file 4 [file Image_2.jpg]

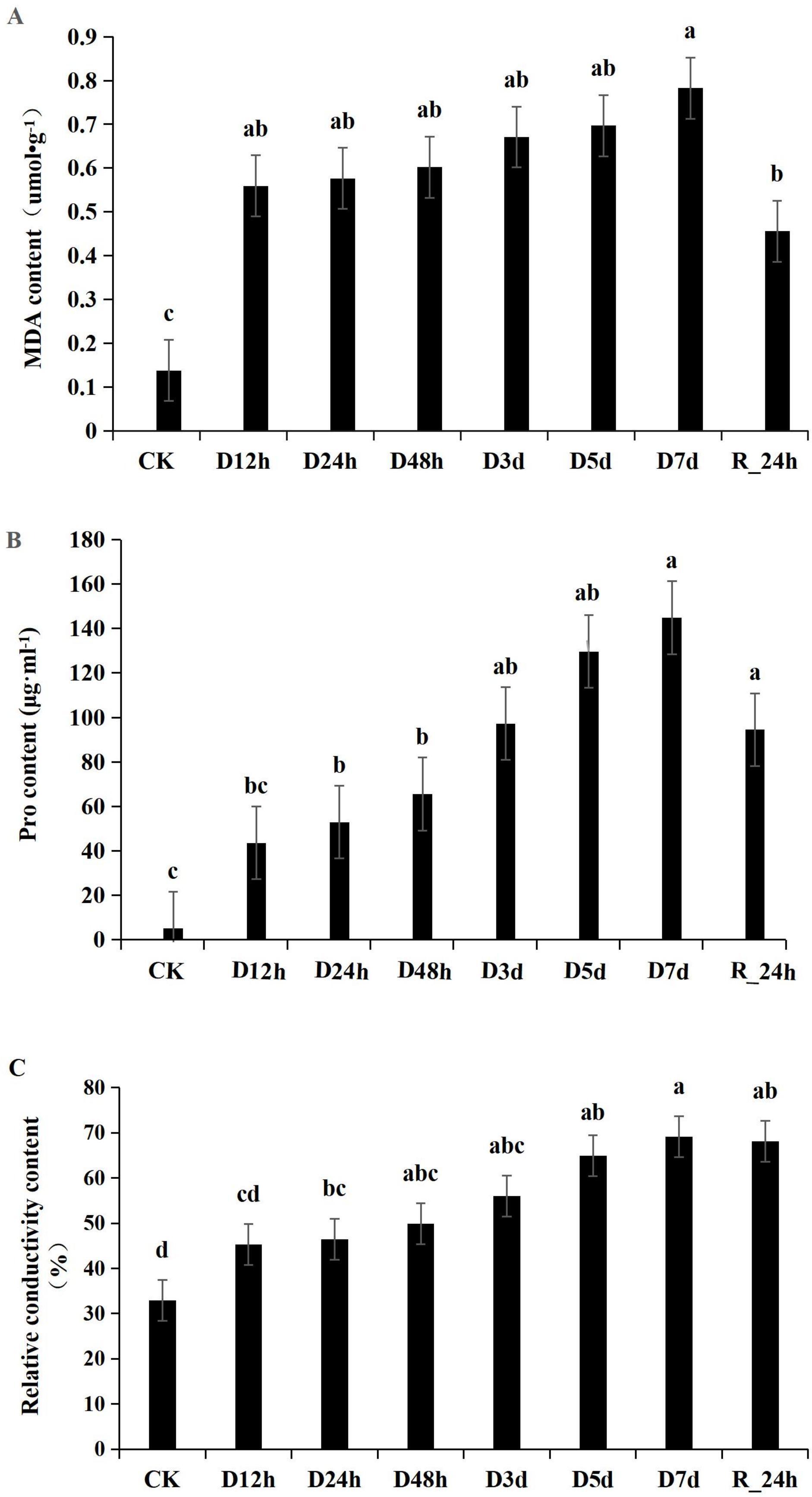

Supplement: Supplementary file 5 [file Image_3.jpg]
