# Supplementary material for: Integrated analysis of small RNAs, transcriptome and degradome sequencing reveal the drought stress network in Agropyron mongolicum Keng
Source: Front Plant Sci. 2022 Aug 17;13:976684. doi: 10.3389/fpls.2022.976684 (PMC9433978; doi:10.3389/fpls.2022.976684)
Supplement: Supplementary file 6 [file Table_1.doc]

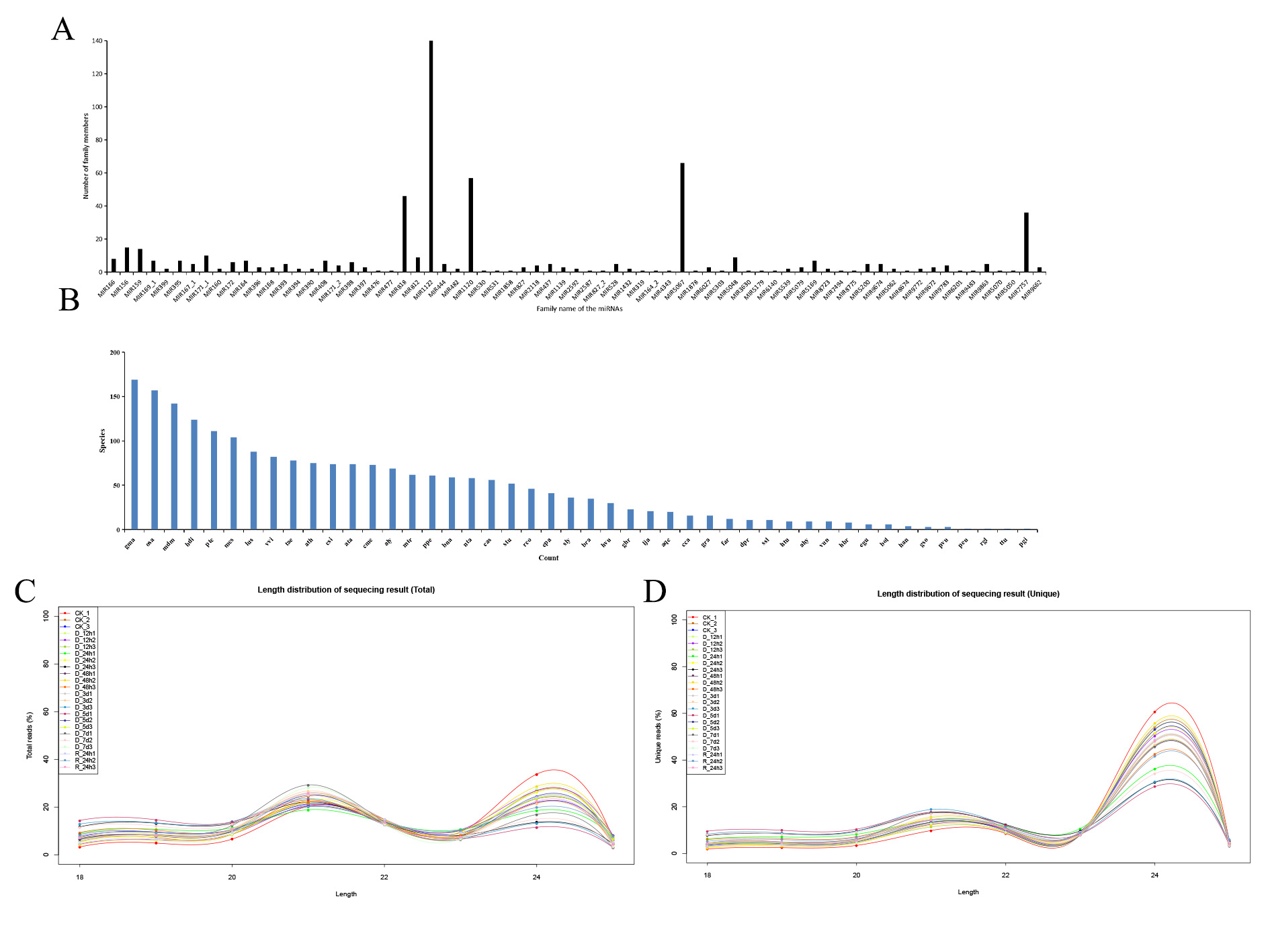


**Supplementary Figure 2 |** Conservation analysis and Family members of miRNAs **(A, B)** and **(C, D)** Length distribution of total reads and unique reads.
